# Leukocyte cell-derived chemotaxin 2 is an antiviral regulator acting through the proto-oncogene MET

**DOI:** 10.1038/s41467-022-30879-3

**Published:** 2022-06-08

**Authors:** Takayoshi Shirasaki, Satoshi Yamagoe, Tetsuro Shimakami, Kazuhisa Murai, Ryu Imamura, Kiyo-Aki Ishii, Hiroaki Takayama, Yukako Matsumoto, Natsumi Tajima-Shirasaki, Naoto Nagata, Ryogo Shimizu, Souma Yamanaka, Atsushi Abe, Hitoshi Omura, Kazunori Kawaguchi, Hikari Okada, Taro Yamashita, Tomoki Yoshikawa, Kazuhiro Takimoto, Motoko Taharaguchi, Shogo Takatsuka, Yoshitsugu Miyazaki, Toshikatsu Tamai, Yamato Tanabe, Makoto Kurachi, Yasuhiko Yamamoto, Shuichi Kaneko, Kunio Matsumoto, Toshinari Takamura, Masao Honda

**Affiliations:** 1 Department of Clinical Laboratory Medicine, Kanazawa University Graduate School of Medical Science, Kanazawa, Japan; 2 Lineberger Comprehensive Cancer Center, The University of North Carolina at Chapel Hill, Chapel Hill, NC USA; 3 Department of Chemotherapy and Mycoses, National Institute of Infectious Diseases, Tokyo, Japan; 4 Department of Gastroenterology, Kanazawa University Graduate School of Medical Sciences, Kanazawa, Japan; 5 Cancer Research Institute, Kanazawa University, Kanazawa, Japan; 6 Institute for Frontier Science Initiative, Kanazawa University, Kanazawa, Japan; 7 Department of Endocrinology and Metabolism, Kanazawa University Graduate School of Medical Sciences, Kanazawa, Japan; 8 Department of Cellular and Molecular Function Analysis, Kanazawa University Graduate School of Medical Science, Kanazawa, Japan; 9 Department of Virology I, National Institute of Infectious Diseases, Tokyo, Japan; 10 Management Department of Biosafety and Laboratory Animal, National Institute of Infectious Diseases, Tokyo, Japan; 11 Department of Molecular Genetics, Kanazawa University Graduate School of Medical Science, Kanazawa, Japan; 12 Department of Biochemistry and Molecular Vascular Biology, Kanazawa University Graduate School of Medical Science, Kanazawa, Japan; 13 WPI-Nano Life Science Institute (WPI-NanoLSI), Kanazawa University, Kanazawa, Japan

**Keywords:** Viral infection, Viral host response

## Abstract

Retinoic acid-inducible gene (RIG)-I is an essential innate immune sensor that recognises pathogen RNAs and induces interferon (IFN) production. However, little is known about how host proteins regulate RIG-I activation. Here, we show that leukocyte cell-derived chemotaxin 2 (LECT2), a hepatokine and ligand of the MET receptor tyrosine kinase is an antiviral regulator that promotes the RIG-I-mediated innate immune response. Upon binding to MET, LECT2 induces the recruitment of the phosphatase PTP4A1 to MET and facilitates the dissociation and dephosphorylation of phosphorylated SHP2 from MET, thereby protecting RIG-I from SHP2/c-Cbl-mediated degradation. In vivo, LECT2 overexpression enhances RIG-I-dependent IFN production and inhibits lymphocytic choriomeningitis virus (LCMV) replication in the liver, whereas these changes are reversed in LECT2 knockout mice. Forced suppression of MET abolishes IFN production and antiviral activity in vitro and in vivo. Interestingly, hepatocyte growth factor (HGF), an original MET ligand, inhibits LECT2-mediated anti-viral signalling; conversely, LECT2-MET signalling competes with HGF-MET signalling. Our findings reveal previously unrecognized crosstalk between MET-mediated proliferation and innate immunity and suggest that targeting LECT2 may have therapeutic value in infectious diseases and cancer.

## Abstract (web summary)

The innate antiviral immune response is an important defense against infection. Here, the authors show that leukocyte cell-derived chemotaxin 2 (LECT2) promotes RIG-I-mediated innate immune responses by preventing its degradation, and inhibits lymphocytic choriomeningitis virus replication in the liver.

## Introduction

Retinoic acid-inducible gene I (RIG-I) is a key sensor of virus recognition and establishes an antiviral host response^1^. The ligands of RIG-I are 5′ tri-phosphorylated RNAs and di-phosphorylated single-stranded RNAs. Once activated, RIG-I induces different signalling pathways that lead to the induction of type I interferons (IFNs) and proinflammatory cytokines^2^. Recently, various studies have suggested that RIG-I plays an important role in not only defence against viral infection but also defence against cancer and autoimmune diseases^3–7^. Therefore, understanding the biology of RIG-I, and in particular the mechanism of RIG-I activation, has important implications for maintaining human health and overcoming disease. Previously, we found that selenoprotein P mRNA, which encodes a liver-derived secretory protein (hepatokine), inhibits type I IFN responses by limiting the function of RIG-I^8^. Recent studies have also suggested that viral infection can regulate RIG-I activation by host cellular RNAs, e.g. non-coding RNAs, instead of viral RNAs^9,10^. However, little is known about host proteins activate the RIG-I pathway.

In this study, we focused on another hepatokine, leukocyte cell-derived chemotaxin 2 (LECT2). LECT2 is a secreted protein originally identified as a chemotactic factor for neutrophils^11^ that is produced predominantly in the liver^12^. LECT2 is now known to be involved in cell development as well as several pathological conditions including sepsis, liver disease, metabolic syndrome and cancer^13–16^. However, the role of LECT2 in innate immune responses and the underlying mechanisms remain largely unclear. The receptors for LECT2 vary from cell to cell and are known to interact with CD209 in dendritic cells, tyrosine kinase with immunoglobulin-like and EGF-like domains 1 in endothelial cells, and MET in hepatocytes^17–19^. MET is a tyrosine kinase receptor with an extracellular α-chain and transmembrane β-chain joined by disulfide bonds^20^. Hepatocyte growth factor (HGF) is a ligand of MET, and HGF/MET signalling regulates cell motility and proliferation in normal conditions^20^. In contrast, inappropriate HGF/MET signalling promotes the onset, proliferation, invasion and metastasis of cancer^21,22^. LECT2 is also a ligand of MET^19^; however, it is unknown whether MET has a role in innate immune responses or if LECT2 affects MET signalling and innate immune responses. Thus, little is known about which host factors are involved in the crosstalk between proliferation and innate immunity.

Here, we demonstrate that LECT2 has a critical role in innate immune responses. Specifically, LECT2 significantly enhances IFN expression in a RIG-I dependent manner through MET. The proliferation signal of HGF and the innate immune response signal of LECT2 mutually regulate each other by using MET as a receptor. MET-mediated crosstalk between LECT2 and HGF. LECT2 may serve as a potential therapeutic target not only for viral infection but also for cancer.

## Results

### LECT2 is a positive regulator of RIG-I-mediated innate immune responses

**Fig. 1:**
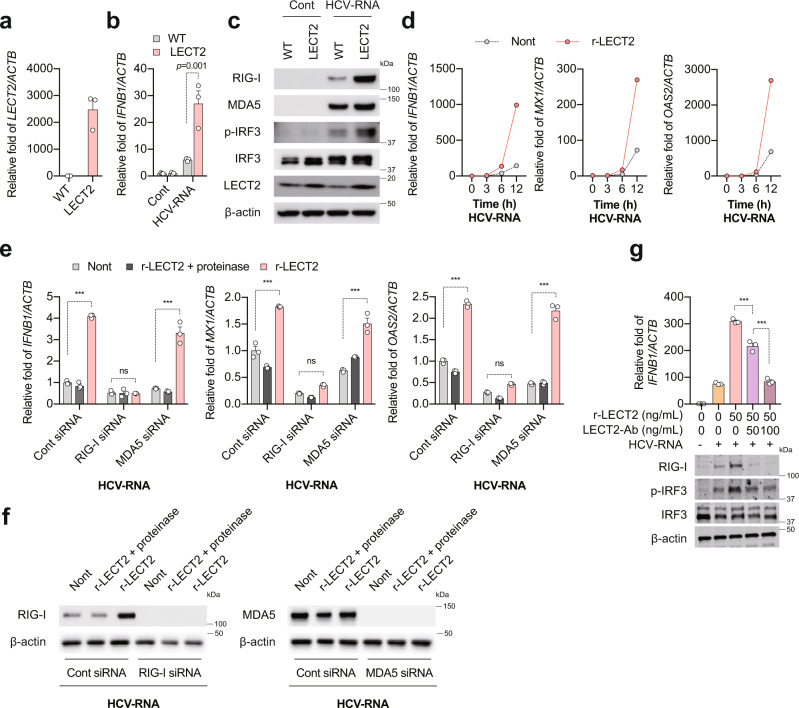
LECT2 is a positive regulator of RIG-I-mediated innate immune responses. **a** qRT-PCR analysis of *LECT2* mRNA in HepG2-WT and HepG2-LECT2 cells. Results were normalised to those of *ACTB*. **b** qRT-PCR analysis of *IFNB1* mRNA in HepG2-WT and HepG2-LECT2 cells at 12 h after mock (Cont) or HCV-RNA transfection. Data shown are means of 3 technical replicates ± SEM. *p* = 0.001 by two-way ANOVA with Tukey’s multiple comparison test. **c** Immunoblot analysis of RIG-I, MDA5, p-IRF3, IRF3, LECT2 and β-actin in HepG2-WT and HepG2-LECT2 cells at 6 h after mock (Cont) or HCV-RNA transfection. **d** qRT-PCR analysis of *IFNB1*, *MX1* and *OAS2* in non-treated (Nont) and r-LECT2-treated HepG2 cells at 0, 3, 6 and 12 h after HCV-RNA transfection. Data shown are means of 2 technical replicates. **e** qRT-PCR analysis of *IFNB1*, *MX1* and *OAS2* in non-treated (Nont), proteinase-treated r-LECT2 (r-LECT2 + proteinase), and r-LECT2-treated cells (Cont siRNA-transfected HepG2 cells, RIG-I siRNA-transfected HepG2 cells, and MDA5 siRNA-transfected HepG2 cells) at 12 h after HCV-RNA transfection. Data shown are means of 3 technical replicates ± SEM. ***p < 0.001, not significant (ns) by two-way ANOVA with Tukey’s multiple comparison test. **f** Immunoblot analysis of RIG-I, MDA5, and β-actin in Nont, r-LECT2 + proteinase and r-LECT2-treated cells at 12 h after HCV-RNA transfection. **g** 50 ng/mL r-LECT2 and LECT2 specific antibody (0, 50 and 100 ng/mL) were reacted for 15 min at room temperature, and then their reactants were treated with HepG2 cells for 1 h and then transfected with HCV-RNA. (upper) qRT-PCR analysis of *IFNB1* at 12 h after HCV-RNA transfection. Data shown are means of 3 technical replicates ± SEM. ***p < 0.001 by two-way ANOVA with Tukey’s multiple comparison test. (lower) Immunoblot analysis of RIG-I, MDA5, p-IRF3, IRF3, LECT2 and β-actin at 12 h after HCV-RNA transfection. Source data are provided as a Source data file.

To examine the functional relevance of LECT2 in viral infection, we evaluated *LECT2*, *MET* and *HGF* mRNA expression in the liver of patients with chronic hepatitis C (CHC). *LECT2* mRNA was significantly up-regulated in the early stage of CHC (F12) compared with patients with simple steatosis, although *LECT2* mRNA expression was decreased in the advanced stage of CHC (F34) (Supplementary Fig. 1). Similarly, *MET* mRNA was up-regulated in CHC (F12) and decreased in the advanced stage of CHC (F34) (Supplementary Fig. 1). Conversely, *HGF* mRNA was up-regulated in CHC (F12) and was further up-regulated in the advanced stage of CHC (F34) (Supplementary Fig. 1). *LECT2* mRNA was significantly correlated with *MET* mRNA, while *HGF* mRNA was not correlated with *MET* mRNA (Supplementary Fig. 1), suggesting the interaction between LECT2 and MET has biological significance in CHC. In primary human hepatocytes, *LECT2* mRNA was induced by 4-5-fold following JFH-1^23^ infection (reference data GSE31264 and GSE31455); therefore, LECT2 might be involved in the regulation of hepatitis C virus (HCV) replication and the pathogenesis of CHC. To determine the effect of LECT2 on innate immune responses in hepatocytes, we established a human hepatocellular carcinoma cell line overexpressing LECT2 (HepG2-LECT2 cells) by using a lentivirus expression system. The abundant expression of *LECT2* mRNA and protein was observed in HepG2-LECT2 cells (Fig. 1a, c). Next, HepG2 and HepG2-LECT2 cells were transfected with HCV-RNA or Poly(I:C). LECT2 overexpression significantly enhanced HCV-RNA- and Poly(I:C)-induced *IFNB1* expression (Fig. 1b and Supplementary Fig. 2a). LECT2 overexpression enhanced HCV-RNA- and Poly(I:C)-induced *RIG-I* expression, but not melanoma differentiation-associated protein 5 (*MDA5*) expression (Fig. 1c and Supplementary Fig. 2b). HCV-RNA- and Poly(I:C)-induced IFN regulatory factor 3 (IRF3) phosphorylation was also significantly higher in HepG2-LECT2 cells than in HepG2 cells (Fig. 1c and Supplementary Fig. 2b). Since LECT2 is a secretory protein, we prepared recombinant LECT2 protein (r-LECT2). Consistent with the results of HepG2-LECT2 cells, the HCV-RNA-induced expression of *IFNB1* and IFN-stimulated genes (ISGs; MX dynamin-like GTPase 1 [*MX1*] and 2′-5′-oligoadenylate synthetase 2 [*OAS2*]) was enhanced in r-LECT2-treated HepG2 cells (Fig. 1d). As RIG-I expression was markedly induced in HepG2-LECT2 cells and r-LECT2-treated HepG2 cells by HCV-RNA or Poly(I:C), we reasoned that LECT2 dominantly regulates RIG-I-mediated innate immune responses. To test our hypothesis, we knocked down RIG-I and MDA5 genes in HepG2 cells by using small interfering RNA (siRNA). RIG-I knockdown completely abolished r-LECT2-activated *IFNB1* and ISG expression, while MDA5 knockdown had no effect on the LECT2 activation phenotype (Fig. 1e, f). To confirm these results in vivo, we generated LECT2 transgenic mice (*Lect2-TG*) and LECT2 knockout mice (*Lect2-KO*) and intravenously injected these mice with Poly(I:C). *Lect2-TG* mice showed a dramatic enhancement of Poly(I:C)-induced *Ifnb1* and ISG (*Mx1* and chemokine [C-X-C motif] ligand 10 [*Cxcl10*]) expression (Supplementary Fig. 3a). In contrast, *Lect2-KO* mice showed a significant impairment of Poly(I:C)-induced *Ifnb1* and ISG expression (Supplementary Fig. 3b). Next, we isolated primary mouse hepatocytes from adult male C57BL/6J mice and treated the cells with recombinant mouse LECT2 protein (r-mouse Lect2), and then transfected the cells with Poly(I:C). Similar to the results obtained with HepG2 cells, r-mouse Lect2-treated primary mouse hepatocytes had enhanced Poly(I:C)-induced *Ifnb1* and ISG (*Mx1* and *Cxcl10*) expression (Supplementary Fig. 3c). However, r-mouse Lect2 did not affect lipopolysaccharide (LPS)-stimulated Toll-like receptor 4 signalling (Supplementary Fig. 3d). Interestingly, r-LECT2-mediated innate immune activation was blocked by LECT2 specific antibody (Fig. 1g). Collectively, these results demonstrated that LECT2 is a positive regulator of RIG-I-mediated innate immune responses.

### LECT2 is an antiviral hepatokine

**Fig. 2:**
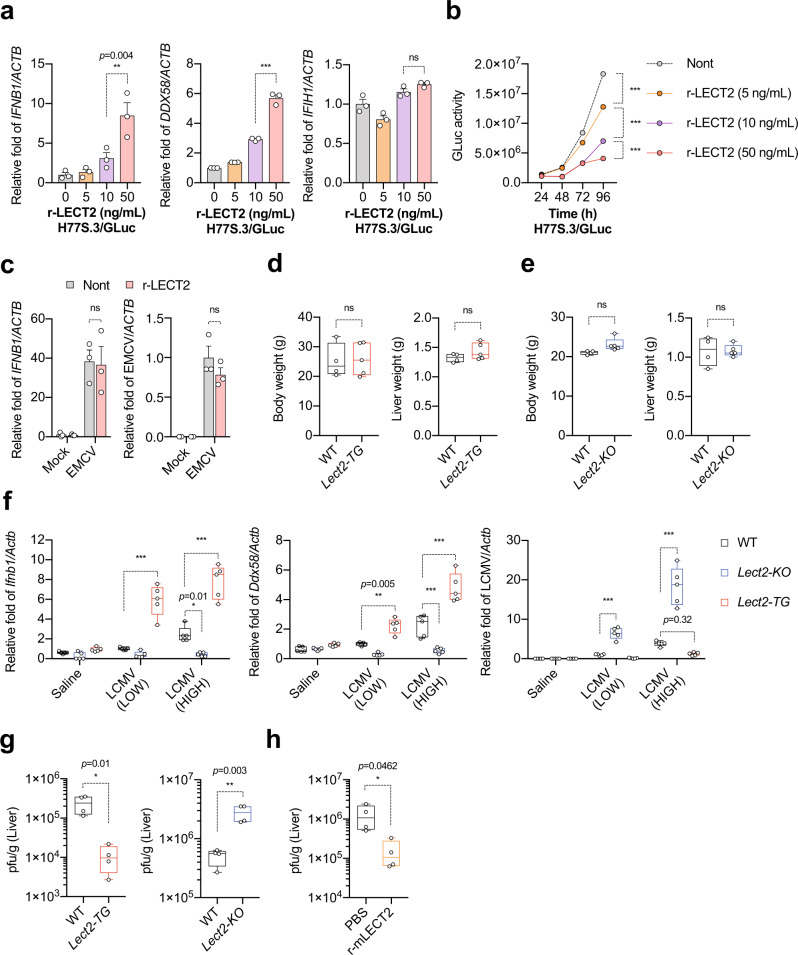
LECT2 is an antiviral hepatokine. **a** qRT-PCR analysis of *IFNB1*, *DDX58* and *IFIH1* in r-LECT2-treated KH cells at 12 h after H77S.3/GLuc-RNA transfection. Results were normalised to those of *ACTB*. Data shown are means of 3 technical replicates ± SEM. **p = 0.004, ***p < 0.001, not significant (ns) by two-way ANOVA with Tukey’s multiple comparison test. **b** GLuc activity in r-LECT2-treated KH cells. Data shown are means of 3 technical replicates ± SEM. ***p < 0.001 by two-way ANOVA with Tukey’s multiple comparison test. **c** qRT-PCR analysis of *IFNB1* and EMCV in non-treated and r-LECT2-treated KH cells at 24 h after EMCV infection. Data shown are means of 3 technical replicates ± SEM. Not significant (ns) by two-way ANOVA with Tukey’s multiple comparison test. **d**, **e**, **f**, **g** WT, *Lect2-TG* and *Lect2-KO* mice were infected with 1.0 × 10^4^ pfu (LOW) or 1.0 × 10^6^ pfu (HIGH) LCMV via intraperitoneal injection. **d**, **e** Body weight and liver weight of these mice before virus infection. Data shown are means of 4 (WT) or 5 (*Lect2-TG* and *Lect2-KO*) mice ± SEM. Not significant (ns) by two-way ANOVA with Tukey’s multiple comparison test. **f** qRT-PCR analysis of *Ifnb1*, *Ddx58* and LCMV in the liver at 7 days after LCMV infection. Data shown are means of 5 biological replicates ± SEM. ***p < 0.001, **p = 0.005, *p = 0.01 by two-way ANOVA with Tukey’s multiple comparison test. **g** Plaque assay in the liver on 7 days post infection. Data shown are means of 4 biological replicates;± SEM. *p = 0.01, **p = 0.003 by two-way ANOVA with Tukey’s multiple comparison test. **h** WT mice (20 g of body weight) were injected with r-mLect2 (50 μg) and then infected with 1.0 × 10^4^ pfu LCMV. Plaque assay in the liver on 6 days post infection. Data shown are means of 4 biological replicates ± SEM. *p = 0.0462 by two-way ANOVA with Tukey’s multiple comparison test. Boxplots: centre line, median; box limits, 25 to 75th percentiles; whiskers, min to max. Source data are provided as a Source data file.

To determine how LECT2 influences antiviral responses in hepatocytes, we used KH cells, which were established from human hepatocellular carcinoma^24^. Although KH cells have an intact RIG-I pathway, they can support the entire life cycle of HCV^24^. KH cells were treated with r-LECT2 and then transfected with synthetic viral RNA (H77S.3/GLuc2A; H77 strain with *Gaussia* luciferase [GLuc] 2A is a construct in which the GLuc sequence, fused to the 2A auto-catalytic protease of foot-and-mouth virus RNA, is inserted in-frame between p7 and NS2)^25^. Similar to the results obtained with HepG2 cells, r-LECT2 treatment of KH cells transfected with H77S.3/GLuc-RNA significantly enhanced *IFNB1* and RIG-I (*DDX58*) expression in a dose-dependent manner, but not MDA5 (*IFIH1*) expression (Fig. 2a). In this setting, r-LECT2 strongly inhibited HCV replication (Fig. 2b). We also established KH cells overexpressing LECT2 (KH-LECT2 cells) by using a lentivirus expression system (Supplementary Fig. 4a, b). KH-LECT2 cells also showed significantly enhanced *IFNB1* and *DDX58* expression, but not *IFIH1* expression (Supplementary Fig. 4c). Consistent with this result, LECT2 overexpression strongly inhibited HCV replication (Supplementary Fig. 4d). As encephalomyocarditis virus (EMCV) is known to be recognised predominantly by MDA5^26–28^, we assessed EMCV replication in r-LECT2-treated KH cells following infection with EMCV at a multiplicity of infection (MOI) of 1.0. As expected, r-LECT2 did not affect EMCV replication or *IFNB1* expression (Fig. 2c). These results suggest that LECT2 has a positive effect on the activation of RIG-I-mediated antiviral responses in hepatocytes. To examine further whether LECT2 is important for innate immune responses against infection with viruses other than HCV, we infected *Lect2-TG* and *Lect2-KO* mice with lymphocytic choriomeningitis virus (LCMV) or mouse hepatitis virus (MHV) via intraperitoneal injection. Body weight and liver weight in *TG* and *KO* mice were not significantly different from those in wild-type (WT) mice (Fig. 2d, e). After infection, *Ifnb1* and *Ddx58* expression was significantly enhanced in *Lect2-TG* mice, whereas their expression was impaired in *Lect2-KO* mice (Fig. 2f and Supplementary Fig. 4e). Consistent with this result, viral load was significantly lower in the liver of *Lect2-TG* mice than in the liver of WT mice (Fig. 2f, g, and Supplementary Fig. 4e). In contrast, viral load was significantly higher in the liver of *Lect2-KO* mice than in the liver of WT mice (Fig. 2f, g, and Supplementary Fig. 4e). Next, we injected WT mice with recombinant mouse LECT2 protein (r-mLect2) via intravenous injection then infected with LCMV. Importantly, viral load was significantly lower in the liver of r-mLect2 treated mice than in the liver of PBS treated mice (Fig. 2h). Collectively, these results revealed that LECT2 activates innate immune responses and suppresses viral replication in hepatocytes.

### MET has an important role in innate immune responses

**Fig. 3:**
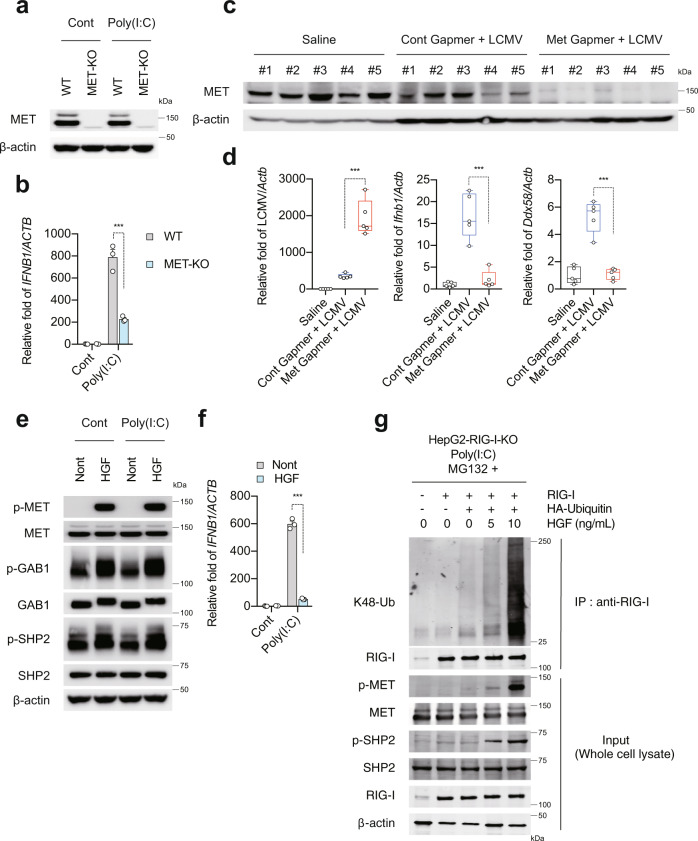
MET has an important role in innate immune responses. **a** Immunoblot analysis of MET and β-actin in HepG2-WT and HepG2-MET-KO cells at 12 h after Cont or Poly(I:C) transfection. **b** qRT-PCR analysis of *IFNB1* in HepG2-WT and HepG2-MET-KO cells at 12 h after Cont or Poly(I:C) transfection. Results were normalised to those of *ACTB*. Data shown are means of 3 technical replicates ± SEM. ***p < 0.001 by two-way ANOVA with Tukey’s multiple comparison test. **c**, **d** C57BL/6J mice were injected intravenously with Cont Gapmer or Met Gapmer, and at 1 day after injection, the mice were infected intraperitoneally with 1.0 × 10^4^ pfu LCMV. **c** Immunoblot analysis of MET and β-actin in the liver of WT mice at 5 days after LCMV infection. **d** qRT-PCR analysis of LCMV, *Ifnb1* and *Ddx58* in the liver of WT mice at 5 days after LCMV infection. Data shown are means of 5 biological replicates ± SEM. ***p < 0.001 by two-way ANOVA with Tukey’s multiple comparison test. Boxplots: centre line, median; box limits, 25 to 75th percentiles; whiskers, min to max. **e** Immunoblot analysis of p-MET, MET, p-GAB1, GAB1, p-SHP2, SHP2 and β-actin in non-treated HepG2 cells and HGF-treated HepG2 cells at 12 h after Cont or Poly(I:C) transfection. **f** qRT-PCR analysis of *IFNB1* in non-treated HepG2 cells and HGF-treated HepG2 cells at 12 h after Cont or Poly(I:C) transfection. Data shown are means of 3 technical replicates ± SEM. ***p < 0.001 by two-way ANOVA with Tukey’s multiple comparison test. **g** HepG2-RIG-I-KO cells were transfected with RIG-I plasmid, HA-ubiquitin plasmid. At 48 h after transfection, the cells were treated with HGF (0, 5 and 10 ng/mL) for 1 h, then transfected with Poly(I:C) in the presence of MG132. At 12 h after Poly(I:C) transfection, lysates were immunoprecipitated with an anti-RIG-I antibody. Immunoblot analysis of K48-Ub, RIG-I, p-MET, MET, p-SHP2, SHP2 and β-actin in immunoprecipitated samples and input cell lysate. Source data are provided as a Source data file.

Next, we explored the mechanism by which LECT2 regulates innate immune responses. MET is a candidate receptor for LECT2^19^. To determine the effect of MET on innate immune responses, we used CRISPR-Cas9 gene editing to eliminate its expression in HepG2 cells. MET expression was detected in HepG2 cells transduced with a nontargeting single guide RNA (sgRNA) with a scrambled sequence, but not in MET-KO cells transduced with MET-specific sgRNA (Fig. 3a). These cells were transfected with Poly(I:C). MET-KO cells showed a significant impairment of Poly(I:C)-induced *IFNB1* expression (Fig. 3b). We also observed the same results in a human liver bile duct carcinoma cell line (HuCCT1 cells) and immortalised human umbilical vein endothelial cell line (HUEhT-2 cells) (Supplementary Fig. 5). Next, we assessed whether these results also occur in vivo. As *Met-KO* mice are embryonically lethal^29^, we used locked nucleic acid (LNA)-Gapmer^30^ to silence Met expression in the mice. C57BL/6J mice were injected intravenously with Gapmer, and at 1 day after injection, they were infected intraperitoneally with LCMV. At 5 days after LCMV infection, Met Gapmer-injected mice showed a significant reduction of Met protein in the liver (Fig. 3c). Similar to the results in HepG2-MET-KO cells, Met Gapmer-injected mice showed a significant impairment of LCMV infection-induced *Ifnb1* and Rig-I (*Ddx58*) expression in the liver (Fig. 3d). Reflecting this result, Met Gapmer-injected mice showed significantly increased LCMV replication in the liver compared to control Gapmer-injected mice (Fig. 3d). Next, we examined whether HGF, a high-affinity ligand for MET, affects innate immune responses. As expected, HGF induced MET phosphorylation and the activation of GRB2-associated binding protein 1 (GAB1) and SHP2, which are located downstream from MET (Fig. 3e). Interestingly, HGF-treated HepG2 cells showed significant impairment of Poly(I:C)-induced *IFNB1* expression (Fig. 3f). More importantly, we found that K48-linked ubiquitination and degradation of RIG-I were induced by HGF (Fig. 3g). These results suggest that MET itself has an important role in the activation of innate immune responses, but HGF-MET signalling has an immune suppressive role.

### MET is essential for LECT2-mediated innate immune responses

**Fig. 4:**
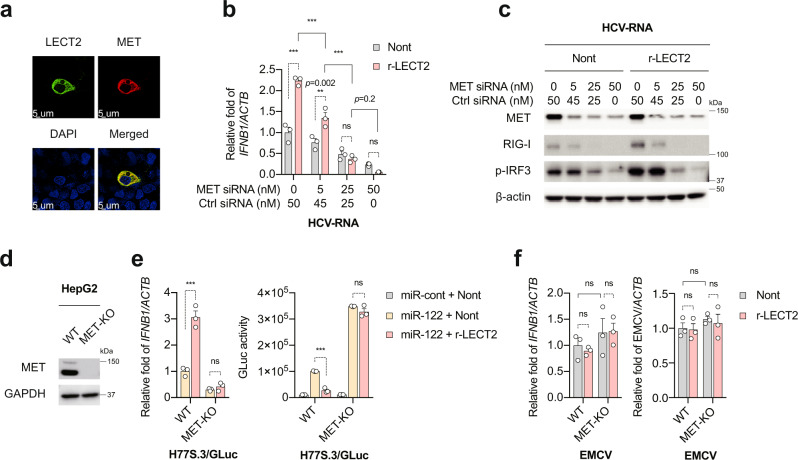
MET is essential for LECT2-mediated innate immune responses. **a** HepG2 cells were transfected with FLAG-tagged LECT2 plasmid and MET plasmid. Co-immunofluorescence staining of LECT2 using an anti-FLAG antibody and MET using an anti-MET antibody in HepG2 cells. DAPI was used to stain nuclei. **b** qRT-PCR analysis of *IFNB1* in MET-knockdown HepG2 cells at 12 h after HCV-RNA transfection in the presence or absence of r-LECT2. Results were normalised to those of *ACTB*. Data shown are means of 3 technical replicates ± SEM. ***p < 0.001, **p = 0.002, not significant (ns) by two-way ANOVA with Tukey’s multiple comparison test. **c** Immunoblot analysis of MET, RIG-I, p-IRF3 and β-actin in MET-knockdown HepG2 cells at 12 h after HCV-RNA transfection in the presence or absence (Nont) of r-LECT2. **d** Immunoblot analysis of MET and GAPDH in HepG2-WT and HepG2-MET-KO cells. **e** (left) qRT-PCR analysis of *IFNB1* in HepG2-WT and HepG2-MET-KO cells at 12 h after miR-122 and H77S.3/GLuc-RNA co-transfection in the presence or absence (Nont) of r-LECT2. Data shown are means of 3 technical replicates ± SEM. ***p < 0.001, not significant (ns) by two-way ANOVA with Tukey’s multiple comparison test. **e** (right) GLuc activity in HepG2-WT and HepG2-MET-KO cells at 12 h after miR-control (miR-cont) or miR-122 and H77S.3/GLuc-RNA co-transfection in the presence or absence (Nont) of r-LECT2. Data shown are means of 3 technical replicates ± SEM. ***p < 0.001, not significant (ns) by two-way ANOVA with Tukey’s multiple comparison test. **f** qRT-PCR analysis of *IFNB1* and EMCV in HepG2-WT and HepG2-MET-KO cells at 24 h after EMCV infection in the presence or absence (Nont) of r-LECT2. Data shown are means of 3 technical replicates ± SEM. Not significant (ns) by two-way ANOVA with Tukey’s multiple comparison test. Source data are provided as a Source data file.

Since LECT2 binds directly to the MET receptor at the cell membrane^19^, we reasoned that the positive regulation of innate immune responses by LECT2 is probably mediated via MET signalling. Indeed, LECT2 co-localised with MET at the HepG2 cell membrane (Fig. 4a). Interestingly, r-LECT2-mediated innate immune activation was completely blocked by depletion of MET protein by specific siRNA (Fig. 4b, c). To determine further whether MET is essential for LECT2-mediated innate immune activation, we used HepG2-MET-KO cells (Fig. 4d) as described earlier. We assessed HCV replication in these cells by co-transfection with synthetic viral RNA (H77S.3/GLuc2A) and the duplex microRNA miR-122^31^, an essential HCV host factor that HepG2 cells lack. HCV replication was enhanced in HepG2-MET-KO cells relative to HepG2-WT cells (Fig. 4e). Consistent with the previous results, HCV replication was suppressed in HepG2-WT cells by treatment with r-LECT2. However, r-LECT2 did not suppress HCV replication in HepG2-MET-KO cells (Fig. 4e). *IFNB1* expression was significantly lower in HCV-RNA-transfected HepG2-MET-KO cells than in HepG2-WT cells (Fig. 4e). Moreover, MET deficiency impaired r-LECT2-induced *IFNB1* expression (Fig. 4e). We obtained similar results with KH-MET-KO cells (Supplementary Fig. 6). Next, we assessed EMCV replication in r-LECT2-treated HepG2-MET-KO cells by infection with EMCV at a MOI of 1.0. MET deficiency did not affect EMCV replication or *IFNB1* expression in the presence or absence of r-LECT2 (Fig. 4f). As LECT2 also binds to the DC-SIGN receptor at the cell membrane^17^, we examined the effect of LECT2 on DC-SIGN signalling, and found that DC-SIGN expression was undetectable in HepG2 cells and r-LECT2 did not affect *IFNB1* and ISG (*MX1* and *OAS2*) expression in DC-SIGN-overexpressing HepG2 cells following HCV-RNA transfection (Supplementary Fig. 7). These results suggest that the interaction of LECT2 and MET is essential for LECT2-mediated innate immune activation, and LECT2 and MET are both indispensable for RIG-I dependent antiviral activity in hepatocytes.

### LECT2 activates innate immune responses by suppressing Tyr1349 phosphorylation in MET

**Fig. 5:**
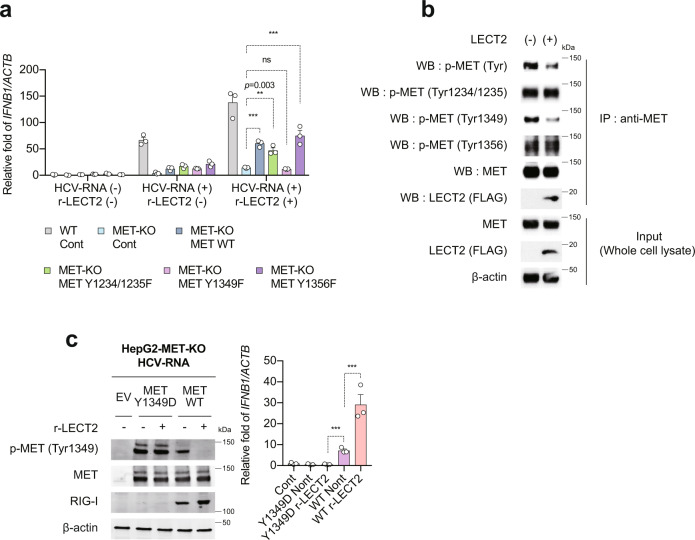
LECT2 activates innate immune responses by suppressing Tyr1349 phosphorylation in MET. **a** HepG2-MET-KO cells were transfected with Control plasmid (Cont), MET WT plasmid (MET WT), or MET mutant plasmid (MET Y1234/1235F, MET Y1349F or MET Y1356F). At 24 h after transfection, the cells were treated with 50 ng/mL r-LECT2 for 1 h and then transfected with HCV-RNA (H77S.3/GLuc-RNA). As a control, HepG2-WT cells were transfected with Cont plasmid; at 24 h after transfection, the Cont plasmid-transfected HepG2-WT cells were treated with 50 ng/mL r-LECT2 for 1 h and then transfected with HCV-RNA. qRT-PCR analysis of *IFNB1* in these cells as described above at 12 h after HCV-RNA transfection. Results were normalised to those of *ACTB*. Data shown are means of 3 technical replicates ± SEM. ***p < 0.001, **p = 0.003, not significant (ns) by two-way ANOVA with Tukey’s multiple comparison test. **b** HepG2 cells were transfected with Control plasmid (LECT2 (−)) or FLAG-tagged LECT2 plasmid (LECT2 ( + )). At 48 h after transfection, lysates were immunoprecipitated with an anti-MET antibody. Immunoblot analysis of p-MET (Tyr), p-MET (Tyr1234/1235), p-MET (Tyr1349), p-MET (Tyr1356), MET, LECT2 (FLAG) and β-actin in immunoprecipitated samples and input cell lysate. **c** HepG2-MET-KO cells were transfected with Control plasmid (EV), MET Y1349D plasmid, or MET WT plasmid. At 24 h after transfection, the cells were treated with 50 ng/mL r-LECT2 for 1 h and then transfected with HCV-RNA (H77S.3/GLuc-RNA). **c** (left) Immunoblot analysis of p-MET (Tyr1349), MET, RIG-I and β-actin at 12 h after HCV-RNA transfection. **c** (right) qRT-PCR analysis of *IFNB1* in these cells as described above at 12 h after HCV-RNA transfection. Results were normalised to those of *ACTB*. Data shown are means of 3 technical replicates ± SEM. ***p < 0.001 by two-way ANOVA with Tukey’s multiple comparison test. Source data are provided as a Source data file.

MET has four tyrosine phosphorylation sites: p-Tyr1003, p-Tyr1234/1235, p-Tyr1349 and p-Tyr1356^20^. To determine how LECT2 affects each phosphorylation site of MET, we employed a set of MET mutants (Y1234/1235F, Y1349F and Y1356F) and transfected these mutants into HepG2-MET-KO cells. Interestingly, partial recovery of r-LECT2-mediated activation of *IFNB1* expression was observed in MET-WT-, Y1234/1235F- and Y1356F-transfected HepG2-MET-KO cells by HCV-RNA transfection. However, Y1349F-transfected HepG2-MET-KO cells could not mediate r-LECT2-mediated innate immune activation (Fig. 5a). Consistent with these results, LECT2 selectively suppressed Tyr1349 phosphorylation in MET (Fig. 5b). Furthermore, we generated a phospho-mimetic (Tyr-to-Asp, Y-D) mutant (MET Y1349D) in which the 1349th tyrosine phosphorylation site of MET is constantly phosphorylated. In MET Y1349D overexpressing cells, dephosphorylation of MET 1349 by LECT2 did not occur as seen in MET WT expressing cells (Fig. 5c). As a result, induction of *IFNB1* expression by HCV-RNA did not occur at all in MET Y1349D overexpressing cells (Fig. 5c). Collectively, these results revealed that LECT2 activates innate immune responses by suppressing Tyr1349 phosphorylation in MET.

### SHP2 inactivation by LECT2 is essential for LECT2-mediated RIG-I dependent innate immune activation

**Fig. 6:**
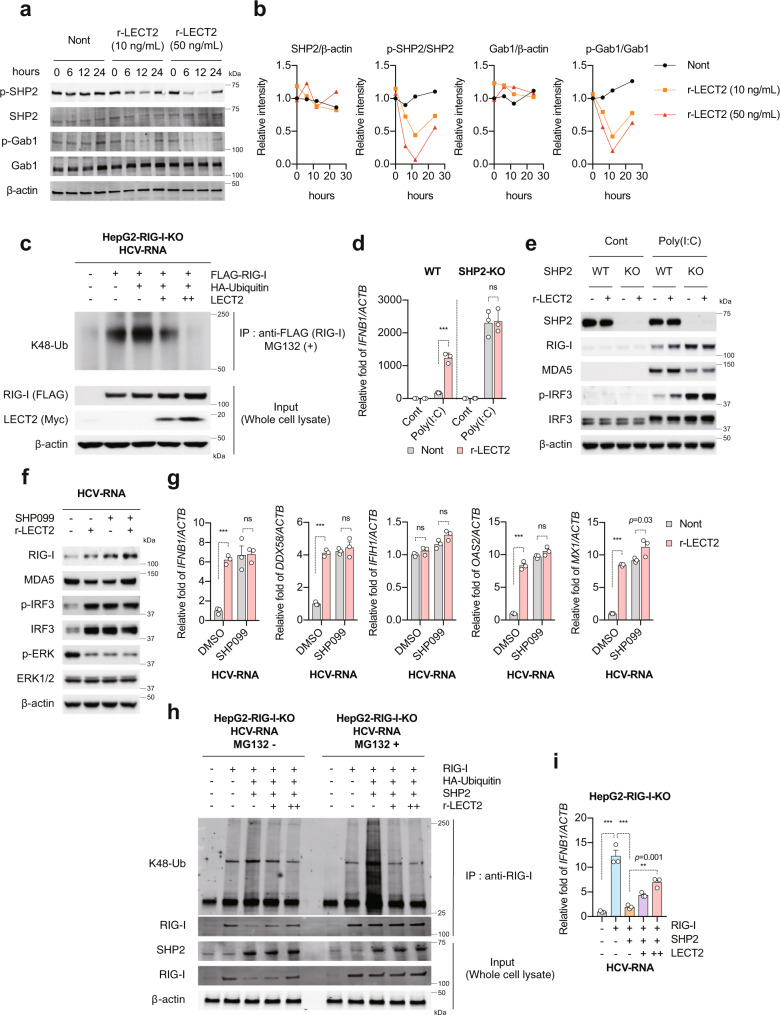
SHP2 inactivation by LECT2 is essential for LECT2-mediated RIG-I dependent innate immune activation. **a** HepG2 cells were treated with r-LECT2. Immunoblot analysis of p-SHP2, SHP2, p-GAB1, GAB1 and β-actin in r-LECT2-treated HepG2 cells. **b** Relative intensity. **c** Immunoblot analysis of K48-ubiquitin (Ub), RIG-I (FLAG), LECT2 (Myc) and β-actin in immunoprecipitated samples and input cell lysate. **d** qRT-PCR analysis of *IFNB1* in HepG2-WT and HepG2-SHP2-KO cells at 12 h after Cont or Poly(I:C) transfection in the presence or absence of r-LECT2. Results were normalised to those of *ACTB*. Data shown are means of 3 technical replicates ± SEM. ***p < 0.001, not significant (ns) by two-way ANOVA with Tukey’s multiple comparison test. **e** Immunoblot analysis of SHP2, RIG-I, MDA5, p-IRF3, IRF3 and β-actin in HepG2-WT and HepG2-SHP2-KO cells at 12 h after Cont or Poly(I:C) transfection in the presence or absence of r-LECT2. **f** Immunoblot analysis of RIG-I, MDA5, p-IRF3, IRF3, p-ERK, ERK1/2 and β-actin in DMSO-treated HepG2 cells and SHP099-treated HepG2 cells at 12 h after HCV-RNA transfection in the presence or absence of r-LECT2. **g** qRT-PCR analysis of *IFNB1*, *DDX58*, *IFIH1*, *OAS2* and *MX1* in DMSO-treated HepG2 cells and SHP099-treated HepG2 cells at 12 h after HCV-RNA transfection in the presence or absence of r-LECT2. Data shown are means of 3 technical replicates ± SEM. ***p < 0.001, not significant (ns) by two-way ANOVA with Tukey’s multiple comparison test. **h** Immunoblot analysis of K48-Ub, SHP2, LECT2 (FLAG), RIG-I and β-actin in immunoprecipitated samples and input cell lysate. **i** HepG2-RIG-I-KO cells were transfected with RIG-I plasmid, SHP2 plasmid and FLAG-tagged LECT2 plasmid. At 48 h after transfection, the cells were transfected with HCV-RNA. qRT-PCR analysis of *IFNB1* in these cells at 12 h after HCV-RNA transfection. Data shown are means of 3 technical replicates ± SEM. ***p < 0.001, **p = 0.001 by two-way ANOVA with Tukey’s multiple comparison test. Source data are provided as a Source data file.

Tyr1349 phosphorylation in the C-terminal docking site of MET is required for various bio-functions via the recruitment of downstream adaptors such as GAB1 and SHP2^20^. Since SHP2 negatively regulates RIG-I protein levels via K48-linked ubiquitination and degradation of RIG-I^32^, we reasoned that the positive regulation of innate immune responses by LECT2 is probably mediated via the suppression of SHP2 activation. Indeed, LECT2 suppressed GAB1 and SHP2 phosphorylation in a dose-dependent manner (Fig. 6a, b). In order to clarify if LECT2 targets RIG-I, we investigated the function of LECT2 on the stabilisation of RIG-I. RIG-I was co-expressed with HA-ubiquitin and LECT2 or a control in HepG2-RIG-I-KO cells. RIG-I ubiquitination was detected in the setting of RIG-I and HA-ubiquitin transfection. However, LECT2 remarkably decreased RIG-I ubiquitination (Fig. 6c). To further determine whether SHP2 is essential for LECT2-mediated innate immune activation, we used CRISPR-Cas9 gene editing to eliminate its expression in HepG2 and KH cells (HepG2-SHP2-KO and KH-SHP2-KO cells) (Fig. 6d, e and Supplementary Fig. 8a). HepG2-SHP2-KO cells showed significantly enhanced Poly(I:C)-induced *IFNB1* expression compared with HepG2-WT cells (Fig. 6d). However, we observed that r-LECT2 no longer activated innate immune responses in the absence of SHP2 (Fig. 6d, e). These results reflected the replication level of HCV (Supplementary Fig. 8b). We also used the SHP2 allosteric inhibitor SHP099^33^ to determine the importance of SHP2 for LECT2-mediated innate immune activation. SHP099 strongly inhibited ERK phosphorylation (Fig. 6f), and we observed almost the same results obtained in HepG2-SHP2-KO cells (Fig. 6f, g). To confirm whether SHP2 is involved in the stabilization of RIG-I by LECT2, we transfected HepG2-RIG-I-KO cells with RIG-I, HA-ubiquitin, SHP2 and LECT2 expression plasmids. In the SHP2 over expressing cells, K48-linked ubiquitination and degradation of RIG-I were promoted; however, LECT2 protected RIG-I from ubiquitination in a dose-dependent manner (Fig. 6h). Reflecting this result, co-expression of RIG-I and SHP2 in HepG2-RIG-I-KO cells impaired HCV-RNA-induced *IFNB1* expression; however, LECT2 restored the inhibitory effect of SHP2 (Fig. 6i). Collectively, these results revealed that LECT2 acts to stabilize RIG-I protein expression by suppressing SHP2 activation.

### PTP4A1 is essential for the enhancement of innate immune response by LECT2

**Fig. 7:**
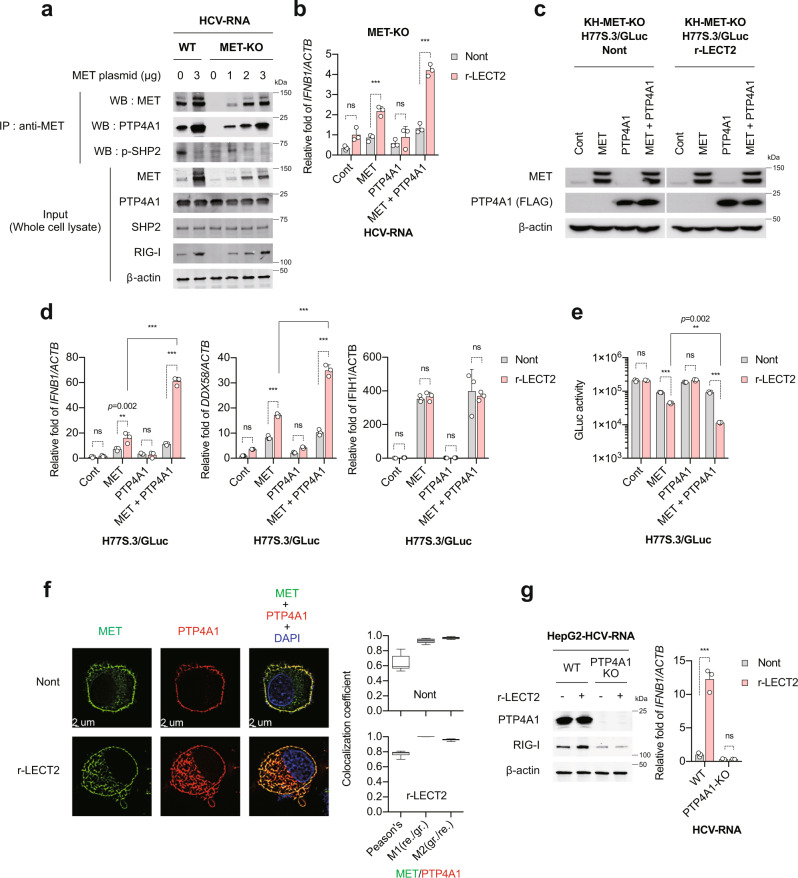
PTP4A1 is essential for the enhancement of innate immune response by LECT2. **a** Immunoblot analysis of MET, PTP4A1, p-SHP2, RIG-I and β-actin in immunoprecipitated samples and input cell lysate. **b** HepG2-MET-KO cells were transfected with Control, MET and PTP4A1 plasmid or co-transfected with MET and PTP4A1 plasmid. These cells were treated with 50 ng/mL r-LECT2 for 1 h and then transfected with HCV-RNA. qRT-PCR analysis of *IFNB1* in these cells. Results were normalised to those of *ACTB*. Data shown are means of 3 technical replicates ± SEM. ***p < 0.001, not significant (ns) by two-way ANOVA with Tukey’s multiple comparison test. **c**–**e** KH-MET-KO cells were transfected with Control, MET and PTP4A1 plasmid or co-transfected with MET and PTP4A1 plasmid. These cells were treated with 50 ng/mL r-LECT2 for 1 h and then transfected with H77S.3/GLuc-RNA. **c** Immunoblot analysis of MET, PTP4A1 and β-actin in these cells. **d** qRT-PCR analysis of *IFNB1*, *DDX58* and *IFIH1* in these cells. Data shown are means of 3 technical replicates ± SEM. ***p < 0.001, **p = 0.002, not significant (ns) by two-way ANOVA with Tukey’s multiple comparison test. **e** GLuc activity in these cells at 48 h after H77S.3/GLuc-RNA transfection. Data shown are means of 3 technical replicates ± SEM. ***p < 0.001, **p = 0.002, not significant (ns) by two-way ANOVA with Tukey’s multiple comparison test. **f** (left) Co-immunofluorescence staining of MET and PTP4A1 in HepG2 cells. DAPI was used to stain nuclei. **f** (right) Co-localization was estimated using Pearson’s correlation coefficient and Manders’ co-localization coefficients M1 (red per green) and M2 (green per red). Boxplots: centre line, median; box limits, 25 to 75th percentiles; whiskers, min to max. **g** (left) Immunoblot analysis of PTP4A1, RIG-I and β-actin at 12 h after HCV-RNA transfection. **g** (right) qRT-PCR analysis of *IFNB1* in HepG2-WT and HepG2-PTP4A1-KO cells at 12 h after HCV-RNA transfection. Data shown are means of 3 technical replicates ± SEM. ***p < 0.001, not significant (ns) by two-way ANOVA with Tukey’s multiple comparison test. Source data are provided as a Source data file.

As LECT2 suppressed MET phosphorylation, we reasoned that LECT2 probably affects the interaction of MET with phosphotyrosine phosphatases (PTPs). We selected a candidate PTP, *PTP4A1*, which was one of the most strongly positively correlated genes with *MET* expression among 30,614 expressed genes in the liver tissue of CHC patients (Supplementary Fig. 1). To test this hypothesis, we immunoprecipitated MET in HepG2-WT and HepG2-MET-KO cells. MET, PTP4A1 and p-SHP2 interactions were detected at the endogenous level in HepG2-WT cells (Fig. 7a). Interestingly, MET overexpression promoted the recruitment of PTP4A1 to MET; however, the recruitment of p-SHP2 was completely abolished (Fig. 7a). The degree of PTP4A1 recruitment to MET was consistent with the expression of endogenous RIG-I (Fig. 7a). This phenomenon was also observed in HepG2-MET-KO cells (Fig. 7a). In the liver tissue of patients with CHC, *LECT2* expression was significantly correlated with *PTP4A1* expression, but not *SHP2* expression. Furthermore, *PTP4A1* expression was significantly correlated with *RIG-I* expression (Supplementary Fig. 1). To determine further whether PTP4A1 recruitment to MET is essential for LECT2-mediated RIG-I dependent innate immune activation, we co-transfected HepG2-MET-KO cells with MET and PTP4A1 expression plasmids. In the absence of MET, PTP4A1 did not enhance r-LECT2-induced *IFNB1* expression (Fig. 7b). However, in the presence of MET, PTP4A1 significantly increased r-LECT2-induced *IFNB1* expression (Fig. 7b). Next, we confirmed this phenomenon by using KH-MET-KO cells (Fig. 7c, d). The same results were obtained in KH-MET-KO cells as in HepG2-MET-KO cells for *IFNB1* and RIG-I (*DDX58*) expression (Fig. 7d). PTP4A1 did not affect MDA5 (*IFIH1*) expression (Fig. 7d). In the presence of MET and PTP4A1, r-LECT2 strongly inhibited HCV replication (Fig. 7e). Next, we performed immunofluorescence staining to confirm PTP4A1 recruitment to MET visually. In the absence of r-LECT2, MET and PTP4A1 co-localization were partially consistent; however, in the presence of r-LECT2, their co-localization was completely consistent in cytoplasm or endosome as well as cell surface (Fig. 7f). Next, we established PTP4A1 knockout cells (PTP4A1-KO cells) and evaluated the effect of LECT2 to enhance RIG-I dependent innate immune response. In the absence of PTP4A1, we found that LECT2 did not enhance the innate immune response at all. This result indicates that PTP4A1 is essential for the enhancement of innate immune response by LECT2 (Fig. 7g).

### PTP4A1 recruitment to MET is essential for LECT2-mediated RIG-I dependent innate immune responses

**Fig. 8:**
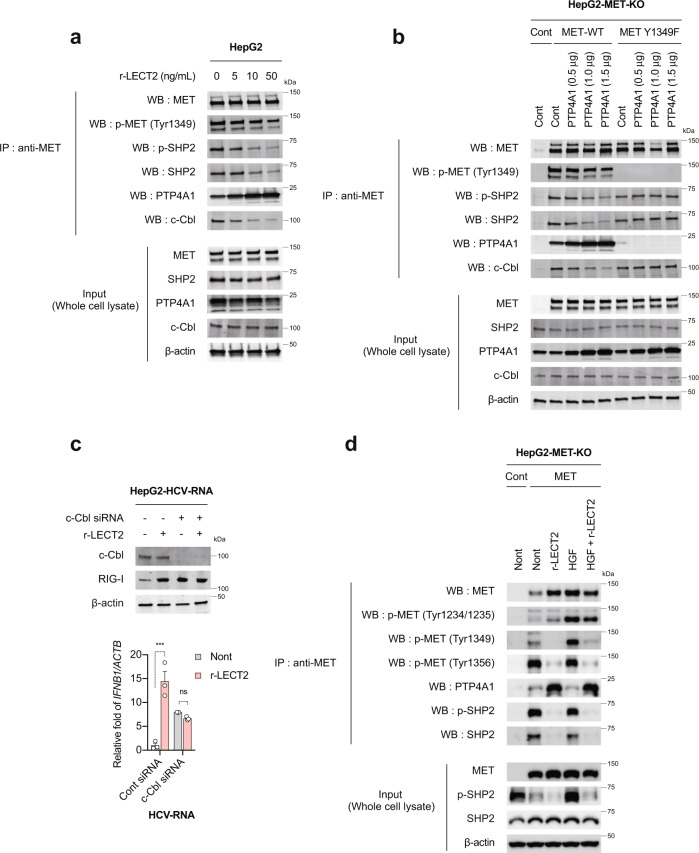
PTP4A1 recruitment to MET is essential for LECT2-mediated RIG-I dependent innate immune responses. **a** HepG2 cells were treated with r-LECT2 (0, 5, 10, 50 ng/mL). At 6 h after treatment, lysates were immunoprecipitated with an anti-MET antibody. Immunoblot analysis of MET, p-MET (Tyr1349), p-SHP2, SHP2, PTP4A1, c-Cbl and β-actin in immunoprecipitated samples and input cell lysate. **b** HepG2-MET-KO cells were transfected with Control plasmid (Cont), MET WT plasmid, MET Y1349F plasmid, or PTP4A1 plasmid. At 48 h after transfection, lysates were immunoprecipitated with an anti-MET antibody. Immunoblot analysis of MET, p-MET (Tyr1349), p-SHP2, SHP2, PTP4A1, c-Cbl and β-actin in immunoprecipitated samples and input cell lysate. **c** HepG2 cells were transfected with 50 nM c-Cbl siRNA. The concentration of siRNA was adjusted to 50 nM with control siRNA. At 24 h after siRNA transfection, siRNA-transfected HepG2 cells were treated with 50 ng/mL r-LECT2 for 1 h and then transfected with HCV-RNA. **c** (upper) Immunoblot analysis of c-Cbl, RIG-I and β-actin at 12 h after HCV-RNA transfection. **c** (lower) qRT-PCR analysis of *IFNB1* at 12 h after HCV-RNA transfection. Results were normalised to those of *ACTB*. Data shown are means of 3 technical replicates ± SEM. ***p < 0.001, not significant (ns) by two-way ANOVA with Tukey’s multiple comparison test. **d** HepG2-MET-KO cells were transfected with Control plasmid or MET plasmid; at 48 h after transfection, the cells were treated with 50 ng/mL r-LECT2 and/or 10 ng/mL HGF. At 1 h after r-LECT2 and/or HGF treatment, lysates were immunoprecipitated with an anti-MET antibody. Immunoblot analysis of MET, p-MET (Tyr1234/1235), p-MET (Tyr1349), p-MET (Tyr1356), PTP4A1, p-SHP2, SHP2 and β-actin in immunoprecipitated samples and input cell lysate. Source data are provided as a Source data file.

The p-SHP2/cCbl complex forms complex with RIG-I and ubiquitinate RIG-I (K48 Ub) that undergoes to proteasome degradation^32^. Indeed, in the physiological ligand-free state, the Tyr1349 phosphorylation site of MET is phosphorylated and binds to the phosphatase PTP4A1 (Fig. 8a). In addition, p-SHP2 and c-Cbl are also bound to MET (Fig. 8a). Interestingly, LECT2 increased the recruitment of PTP4A1 to MET, while decreased the binding of p-SHP2 and c-Cbl to MET (Fig. 8a). Overexpression of PTP4A1 increased the MET associated PTP4A1 and decreased MET associated p-SHP2 and c-Cbl (Fig. 8b). Importantly, p-Tyr1349 mutant (Y1349F) did not recruit PTP4A1 on MET and the levels of MET associated p-SHP2 and c-Cbl did not change (Fig. 8b). The results indicated PTP4A1 targeted p-Tyr1349 and competed with p-SHP2 at p-Tyr1349. We also found that LECT2 did not enhance the innate immune response in c-Cbl2 knockdown cells (Fig. 8c). Finally, we assessed the effect of LECT2 on HGF signalling with p-Tyr1349, p-SHP2 and PTP4A1 expression and found that LECT2 fully competed with HGF signalling (Fig. 8d). Collectively, these results reveal that LECT2 activates RIG-I dependent innate immune responses by promoting the recruitment of PTP4A1 to MET, which suppresses the activation of SHP2/c-Cbl mediated RIG-I ubiquitination.

## Discussion

In the field of infectious immunity, LECT2 protects against *Escherichia coli*, *Pseudomonas aeruginosa* and *Helicobacter pylori* infections by activating macrophages via CD209a^17,34^. However, the regulation of *LECT2* mRNA expression has not been clarified. LECT2 is regulated by liver regeneration^35^ and a recent report showed that *LECT2* mRNA is induced in non-alcoholic fatty liver disease. In this study, we and reference data (GSE31264 and GSE31455) showed that *LECT2* mRNA is induced in cultured cells by HCV infection and significantly up-regulated in the liver tissue of patients with early-stage CHC compared with simple steatosis patients (Supplementary Fig. 1). Furthermore, CHC patients with a sustained virological response showed significantly higher *LECT2* mRNA expression than patients without a sustained virological response (Supplementary Fig. 1). Here, we showed that LECT2 plays a role in anti-viral defence by protecting the SHP2/c-Cbl mediated RIG-I degradation via MET. However, LECT2-MET-mediated innate immune responses may not be restricted only to hepatocytes, and they might also have a role in immune cell activation. In vivo, the increase of LCMV infection by the forced suppression of MET using Gapmer might be explained by the decrease of RIG-I activity in hepatocytes and immune cells.

Mechanistically, we demonstrated that LECT2 activates innate immune responses via the suppression of Tyr1349 phosphorylation in MET. The catalytic docking site (Tyr1234/1235) of MET positively modulates enzyme activity, while its multifunctional docking site (Tyr1349/1356) recruits several transducers and adaptors, e.g. GAB1, SHC, GRB2 and SHP2. Recent studies demonstrated that SHP2/c-Cbl complex promotes K48-linked ubiquitination and proteasomal degradation of RIG-I^32^. These reports are in full conformity with our results showing that HGF promotes SHP2 phosphorylation, leading to the degradation of RIG-I by SHP2/c-Cbl complex and LECT2 promotes SHP2 dephosphorylation, thereby inhibiting RIG-I degradation by SHP2/c-Cbl complex. More importantly, we found that PTP4A1 is a physiological partner of MET that dephosphorylates SHP2 in ligand-free conditions. Depleting MET released PTP4A1 from MET and increased p-SHP2/c-Cbl complex, which promoted RIG-I degradation. In this sense, MET is a physiological immune molecule against pathogens by protecting RIG-I from degradation. We showed that the binding of LECT2 to MET leads to the further recruitment of PTP4A1 to a phosphorylation site of MET, resulting in the dissociation of SHP2 and c-Cbl from MET. Conversely, HGF competes with LECT2 signalling by dissociating PTP4A1 from MET and increasing the recruitment of SHP2. HGF and LECT2 reportedly bind to different portions of the extracellular domain of MET, i.e. HGF binds to the β-chain and LECT2 binds to the α-chain^19^. Therefore, the different signalling pathways of HGF and LECT2 observed in this study could be explained as the binding of HGF and LECT2 at the cell surface changed the conformation of the cytoplasmic domain of MET to open or close the Tyr1349 site for the recruitment of PTP4A1. Further study using protein crystallisation could reveal the detailed mechanism of PTP4A1 recruitment and SHP2 dephosphorylation.

Nevertheless, our data indicated that MET serves as a ‘molecular switch’ by selecting ligands for innate immune signalling and cell proliferation. This mechanism might be beneficial for inhibiting viral proliferation in infected cells and preventing viral spread during hepatitis, and then once the virus is eliminated, it may enable a quick shift from anti-viral immune signalling to hepatocyte regeneration. HGF-MET signalling participates in haematopoietic cell differentiation; therefore, the increased expression of MET in immune cells reported previously^36^ should enable immune cells to switch quickly from a state of differentiation/proliferation to become functional immune effector cells.

LECT2 expression and RIG-I activation are associated with the prognosis of liver cancer^19,37^. However, current MET inhibitors also eliminate the LECT2-dependent innate immune-stimulating effects^38^. Therefore, the development of MET inhibitors which preserve LECT2-MET signalling could be an attractive and effective therapeutic approach.

In conclusion, our studies have revealed a previously unrecognized role for LECT2 in innate immune responses and provide insight into the crosstalk that exists between innate immunity and cell proliferation in the liver (Supplementary Fig. 10). They add to our understanding of the link between infection, tissue repair and cancer.

## Methods

### Study approval and ethics statements

All in vitro studies were carried out with approval from the Ethics Committee at Kanazawa University. All mouse studies were carried out in accordance with the Guidelines for the Care and Use of Laboratory Animals issued by the National Institute of Infectious Diseases. The protocol was approved by the ethics committee of the National Institute of Infectious Diseases.

For human research participants, the research protocols were reviewed by the ethics committee at Kanazawa University and its related hospitals. Informed consent was obtained from all patients.

### Cells

HepG2 cells were purchased from the American Type Culture Collection (Manassas, VA). KH cells were established from surgically resected hepatocellular carcinoma tissue from a patient with CHC^24^. These cells were maintained in Dulbecco’s modified Eagle’s medium (Life Technologies, Carlsbad, CA) containing 10% foetal bovine serum (FBS; Life Technologies), 100 U/mL penicillin and 100 mg/mL streptomycin (Sigma-Aldrich, St. Louis, MO). HuCCT1 cells were kindly provided by Professor Kunio Matsumoto (Kanazawa University, Ishikawa, Japan), and were maintained in RPMI 1640 medium (Life Technologies) containing 10% FBS, 100 U/mL penicillin and 100 mg/mL streptomycin. HUEhT-2 cells were purchased from JCRB (Japanese Collection of Research Bioresources) Cell Bank (National Institutes of Biomedical Innovation, Health and Nutrition, Osaka, Japan), and were maintained in EGM^TM^-2 Endothelial Cell Growth Medium-2 BulletKit^TM^ (Lonza, Basel, Switzerland). Mouse primary hepatocytes were isolated from 8-week-old male C57BL/6J mice as described previously^39^.

### Viruses

EMCV was kindly provided by Professor Mitsutoshi Yoneyama (Chiba University, Chiba, Japan)^26^. LCMV-WE-NIID strain (GenBank accession numbers LC413283 and LC413284) was amplified in BHK-21 cells. MHV-JHM strain was amplified in DBT cells.

### Patients

*LECT2*, *MET*, *HGF*, *PTP4A1*, *SHP2* and *RIG-I* mRNA expression was evaluated using liver tissue obtained from 91 patients with CHC (F12, *n* = 55; F34, *n* = 36) before they received PEG-IFNa-2b (Schering-Plough K.K., Tokyo, Japan) and ribavirin combination therapy^40^, and 30 patients with simple steatosis^8^. The relationship between *LECT2* mRNA expression and treatment efficacy was evaluated in liver tissue from 168 patients with CHC as described previously^41^.

### Mice

The mice were housed in cages with no more than 5 mice per cage and had unrestricted access to autoclaved water and food. All mice were bred and kept under specific pathogen-free conditions. For the generation of LECT2-transgenic mice, the plasmid pSA21 was constructed, which was inserted into the coding region of mouse LECT2 cDNA^42^ into the *Xho*I site of the pCXN2 plasmid^43^. The *Hind*III-*Sal*I fragment of pSA21 was purified by agarose gel electrophoresis and microinjected into the pronucleus of fertilised eggs from C57BL/6J mice. Following microinjection, viable eggs were transferred to the oviducts of pseudopregnant Crj:CD-1 mice. The obtained transgenic mouse line (C57#33) was maintained as hemizygotes by mating with WT mice. The serum concentration of transgenic LECT2 mice was approximately 60 times higher than that of WT mice. The generation of LECT2-knockout mice has been described previously^44^. WT, *Lect2-TG* and *Lect2-KO* mice were all 6-8 week old male mice used in the experiments.

### Reagents and antibodies

Proteinase, saline, dimethyl sulphoxide (DMSO), and MG132 were purchased from Sigma-Aldrich. Poly(I:C), mainly consisting of low molecular weight species (100b–500b > 90%), and LPS was purchased from Sigma-Aldrich. HGF recombinant human protein was purchased from Thermo Fisher Scientific (Waltham, MA). SHP099 was purchased from Cayman Chemical (Ann Arbor, MI). Primary antibodies to RIG-I (#3743), MDA5 (#5321), p-IRF3 (#83611), IRF3 (#4302), p-MET (Tyr1234/1235) (#3077), p-MET (Tyr1349) (#3121), MET (#3127), p-GAB1 (#12745), GAB1 (#3232), p-SHP2 (#5431), SHP2 (#3397), p-ERK (#9101), ERK1/2 (#9102), DC-SIGN (#13193), DYKDDDDK Tag (FLAG) (#14793), Myc (#2276), GAPDH (#2118) and β-actin (ACTB; #4970) were purchased from Cell Signaling Technology (Beverly, MA); phosphotyrosine (05-321) and K48-ubiquitin (05-1307) were from Merck Millipore (Burlington, MA); PRL-1 (PTP4A1) (ab168643) and LECT2 (ab119429) were from Abcam (Cambridge, UK); and p-MET (Tyr1356) (PA5-40218) was from Thermo Fisher Scientific. All antibodies were used at a 1:1000 dilution.

### Plasmids, miRNAs and Gapmer

pH77S.3/GLuc2A has been described previously^25^. The LECT2, PTP4A1 and DC-SIGN cDNA plasmids were purchased from OriGene (Rockville, MD). The HA-ubiquitin plasmid was purchased from Addgene (Watertown, MA). The FLAG-tagged RIG-I plasmid was kindly provided by Professor Takashi Fujita (Kyoto University, Kyoto, Japan). The SHP2 plasmid was kindly provided by Professor Naoto Nagata (Kanazawa University, Ishikawa, Japan). The MET plasmid and MET Y1234/1235F mutant were kindly provided by Professor Kunio Matsumoto (Kanazawa University, Ishikawa, Japan). The MET Y1349F, Y1356F and Y1349D mutants were generated using a QuikChange Site-Directed Mutagenesis Kit (Agilent Technologies, Santa Clara, CA) according to the manufacturer’s protocol by using a forward primer (5′-TTTCATTGGGGAGCACTTTGTCCATGTGAACGCTA-3′) and reverse primer (5′-TAGCGTTCACATGGACAAAGTGCTCCCCAATGAAA-3′) for the Y1349F mutant, forward primer (5′-TCCATGTGAACGCTACTTTTGTGAACGTAAAATGTGTCG-3′) and reverse primer (5′-CGACACATTTTACGTTCACAAAAGTAGCGTTCACATGGA-3′) for the Y1356F mutant, and forward primer (5′-TTTCATTGGGGAGCACGATGTCCATGTGAACGCTA-3′) and reverse primer (5′-TAGCGTTCACATGGACATCGTGCTCCCCAATGAAA-3′) for the Y1349D mutant. miRNA negative control and miR-122 mimic were purchased from Sigma-Aldrich. Plasmids and miRNAs were transfected using Lipofectamine 3000 (Invitrogen, Carlsbad, CA) according to the manufacturer’s instructions. LNA-Gapmer was obtained from QIAGEN (Valencia, CA). The sequence of the Gapmer targeting Met mRNA was 5′-GGATTGGTGAGGTAAT-3′ with LNA.

### Recombinant LECT2 protein

Human LECT2 cDNA in the CCSB Human ORFeome Collection was purchased. Mouse LECT2 cDNA was obtained by RT-PCR using a cDNA library prepared from TAC2 mouse mammary gland epithelial cells. Flag- and His-tag cDNAs were inserted into the 3′-end of human and mouse LECT2 cDNAs. Mammalian expression plasmids for human and mouse LECT2 (pEHX1.1-hLECT2-Flag-His and pEHX1.1-mLECT2-Flag-His) were constructed using the pEHX1.1 vector. pEHX1.1-hLECT2-Flag-His and pEHX1.1-mLECT2-Flag-His linearized by *Ase*I were respectively transfected into CHO-K1 cells using Lipofectamine 3000 (Invitrogen). For selection, the cells were cultured in Ham’s F-12 medium supplemented with 10% FBS and 15 μg/mL puromycin. The cells were cultured in a floating condition at 135 rpm in CHO-CD-XP medium supplemented with 5% FBS, 15 μg/mL puromycin and protein hydrolysate (Hydrolysate Blend XP; Irvine Scientific, Santa Ana, CA) for 6 days in 8% CO_2_, followed by floating culture in the same condition at a larger scale (950 mL) for 8 days. Culture medium was centrifuged at 10,000 × *g* for 10 min and the supernatant was passed through a filter membrane (0.45-μm pore size; Merck Millipore). The medium was applied to a Hi-Trap heparin column (GE Healthcare, Chicago, IL) equilibrated with 50 mM Tris HCl (pH 8.0) buffer containing 0.01% Tween 80, and LECT2 was eluted using a concentration gradient of NaCl in the same buffer. Fractions containing LECT2 were collected, NaCl concentration in the eluate was adjusted to 0.5 M, and applied to a nickel affinity column (cOmplete His-Tag Purification Column; Roche, Mannheim, Germany) equilibrated with 50 mM Tris HCl (pH 8.0) buffer containing 0.01% Tween 80. LECT2 was eluted by a concentration gradient of imidazole in the same buffer (Supplementary Fig. 9). Fractions containing LECT2 were applied and eluted using a heparin column to remove imidazole. Protein concentration was determined by a BCA Protein Assay Kit (Thermo Scientific).

### Establishment of cell lines stably expressing LECT2

A lentiviral transfer plasmid encoding the LECT2 gene was created by PCR by amplifying the LECT2 cDNA plasmid as a template by using a forward primer (5′-TTAGAATTCATGTTTTCCACC-3′) with an *Eco*RI restriction site and a reverse primer (5′-AATTCTAGATTACAGGTATGC-3′) with an *Xba*I restriction site. The PCR product was digested with the above enzymes and ligated into a similarly digested pLVSIN-CMV Pur vector (Takara Bio, Otsu, Japan) to obtain the pLVSIN-CMV-LECT2 vector. Viral particles were generated by transfecting plated Lenti-X 293 T cells (Takara Bio) with the pLVSIN-CMV Pur or pLVSIN-CMV-LECT2 vector, along with Lentiviral High Titer Packaging Mix (Takara Bio) using the FuGENE-HD transfection reagent (Promega, Madison, WI). The supernatant fluids harvested at 72 h were passed through a 0.22-μm syringe filter and transduced into HepG2 and KH cells. At 72 h after transduction, these cells were treated with puromycin (5 mg/mL) for antibiotic selection. We used antibiotic-resistant bulk cell populations for experiments to avoid clonal biases.

### Establishment of MET, SHP2, PTP4A1 and RIG-I KO cell lines

The production of the sgRNA CRISPR/Cas9 lentivirus was carried out by co-transfecting sgRNA-expressing vectors targeting MET, SHP2, PTP4A1 and non-targeting control (Applied Biological Materials, Richmond, Canada) and 3rd Generation Packaging System Mix (Applied Biological Materials) into Lenti-X 293T cells using the FuGENE-HD transfection reagent (Promega). The supernatant fluids harvested at 72 h were passed through a 0.22-μm syringe filter and transduced into HepG2, KH, HuCCT1 and HUEhT-2 cells. At 72 h after transduction, these cells were treated with puromycin (5 mg/mL) for antibiotic selection. We used antibiotic-resistant bulk cell populations for experiments to avoid clonal biases. HepG2-RIG-I-KO cells were described previously^8^.

### RNA interference

siRNAs specific to RIG-I, MDA5 and MET, c-Cbl and Negative Control siRNA were obtained from Sigma-Aldrich. siRNA transfection was performed using Lipofectamine RNAiMAX Transfection Reagent (Invitrogen) according to the manufacturer’s instructions.

### HCV-RNA and Poly(I:C) transfection

In vitro transcription of HCV-RNA (H77S.3/GLuc-RNA) was carried out using the T7 RiboMAX™ Express Large Scale RNA Production System (Promega) according to the manufacturer’s instructions, and synthesised RNA was purified using an RNeasy Mini Kit (Thermo Fisher Scientific). The cells were transfected with HCV-RNA or Poly(I:C) using Lipofectamine 3000 (Invitrogen) according to the manufacturer’s instructions.

### GLuc reporter assay

Cell culture supernatants were harvested at the indicated time points after HCV-RNA transfection and fresh medium was added to the cells. Secreted GLuc was measured by the GloMax-Multi Detection System (Promega) according to the manufacturer’s instructions.

### RNA extraction and quantitative RT-PCR

Total RNA was isolated using a High Pure RNA Isolation Kit (Roche Applied Science, Penzberg, Germany), and cDNA was synthesised with a High Capacity cDNA Reverse Transcription Kit (Applied Biosystems, Foster City, CA). RTD-PCR was performed using the 7900HT Real-Time PCR System (Applied Biosystems) according to the manufacturer’s instructions. The primer pairs and probes for human (*LECT2*, *IFNB1*, *MX1*, *OAS2*, *DDX58*, *IFIH1* and *ACTB*) and mouse (*Ifnb1*, *Ddx58*, *Ifih1*, *Mx1*, *Oas2*, *Cxcl10* and *Actb*) genes were obtained from the TaqMan assay reagents library (Thermo Fisher Scientific). The primers and probe for the quantification of LCMV were forward 5′-CCTGGACTCTGTAATTGGCA-3′, reverse 5′-TTACATGCTCAGCAGCACAG-3′, and probe 5′-FAM-TCACAGTGGATTTCACACACAACCAGA-MGB-3′. The primers and probe for the quantification of MHV were forward 5′-CGGTGTTAGCGGTTTTGCTG-3′, reverse 5′-GCCACTCGGTTTGTTTGAGGGCA-3′, and probe 5′-FAM-TATGTGAAGTCCAAGGTCGGAAATTACCGACTG-MGB-3′. The primers and probe for the quantification of EMCV were forward 5′-GGGATCAGCTTTTACGGCTTT-3′, reverse 5′-TGCATCCGATAGAGAACTTAATGTCT-3′, and probe 5′-FAM-CGATGCCAACGAGGACGCCC-MGB-3′.

### PFU assay

Plaque forming units (PFU) of liver tissue samples from infected mice, were determined by 5-day plaque assay on Vero cell. Cells were plated (70%) confluent at time of infection. Cells were infected with virus for 60 min at 37 °C, and then covered with a 0.5% agarose and complete 1x Medium 199 (Thermo Fisher Scientific) semisolid overlay. Cells were incubated for 5 days, then an overlay of Crystal Violet.

### Sodium dodecyl sulphate-polyacrylamide gel electrophoresis and immunoblotting

The cells were washed in phosphate-buffered saline and lysed in a RIPA Lysis Buffer (EMD Millipore, Burlington, MA) containing complete Protease Inhibitor Cocktail and PhosSTOP (Roche Applied Science). The membranes were blocked in Blocking One or Blocking One-P solution (Nacalai Tesque, Kyoto, Japan). Western blotting was performed with standard methods. The ChemiDoc Imaging System with Image Lab 6.0 software (Bio-Rad, Hercules, CA) was used for visualisation.

### Immunoprecipitation

Immunoprecipitation was performed using Dynabeads Protein G (Invitrogen) according to the protocol of the RIP-Assay Kit (MBL, Nagoya, Japan).

### Immunofluorescence

Cells grown on a 4-well chamber slide were fixed with 4% paraformaldehyde and permeabilized with 0.25% Triton X-100. The cell monolayer was incubated with a mouse anti-MET antibody (Figs. 4a and 6f) and rabbit anti-FLAG antibody (Fig. 4a) or rabbit anti-PTP4A1 antibody (Fig. 6f) for 1 h, followed by a secondary antibody, goat anti-rabbit Alexa Fluor 488 (Fig. 4a) or 594 (Fig. 6f), and goat anti-mouse Alexa Fluor 488 (Fig. 6f) or 594 (Fig. 4a). Nuclei were counterstained with DAPI. Imaging was performed with a Dragonfly CR-DFLY-301 (Oxford Instruments, Oxon UK).

### Statistics and reproducibility

Unless noted otherwise, all between-group comparisons were carried out by one-way or two-way analysis of variance (ANOVA) or a two-sided t-test. Calculations were made using Prism 8 software (GraphPad Software, La Jolla, CA). Replicates of in vivo experiments represent biological replicates, while replicates of in vitro experiments represent technical replicates. Results are representative of three independent experiments.

### Reporting summary

Further information on research design is available in the Nature Research Reporting Summary linked to this article.

## Supplementary information

Supplementary Information

Peer Review File

Reporting Summary

## Data Availability

The data supporting the findings of this study with the article and its supplementary data are included as source data. Any other data that support the findings of this study are available from the corresponding author upon request.

## Source data

Source Data
