# Supplementary material for: Leukocyte cell-derived chemotaxin 2 is an antiviral regulator acting through the proto-oncogene MET
Source: Nat Commun. 2022 Jun 8;13:3176. doi: 10.1038/s41467-022-30879-3 (PMC9177837; doi:10.1038/s41467-022-30879-3)
Supplement: Supplementary file 1 — Supplementary Information [file 41467_2022_30879_MOESM1_ESM.pdf]

Supplementary Information for

Leukocyte cell-derived chemotaxin 2 is an antiviral regulator acting through the proto-oncogene MET

Takayoshi Shirasaki[1,2], Satoshi Yamagoe[3], Tetsuro Shimakami[4], Kazuhisa Murai[1], Ryu Imamura[5,6], Kiyo-Aki Ishii[7], Hiroaki Takayama[7], Yukako Matsumoto[7], Natsumi Tajima-Shirasaki[7], Naoto Nagata[8], Ryogo Shimizu[1], Souma Yamanaka[1], Atsushi Abe[1], Hitoshi Omura[4], Kazunori Kawaguchi[4], Hikari Okada[4], Taro Yamashita[4], Tomoki Yoshikawa[9], Kazuhiro Takimoto[10], Motoko Taharaguchi[10], Shogo Takatsuka[3], Yoshitsugu Miyazaki[3], Toshikatsu Tamai[11], Yamato Tanabe[11], Makoto Kurachi[11], Yasuhiko Yamamoto[12], Shuichi Kaneko[4], Kunio Matsumoto[5,6,13], Toshinari Takamura[7] and Masao Honda[1,4]

[1] Department of Clinical Laboratory Medicine, Kanazawa University Graduate School of Medical Science, Kanazawa, Japan

[2] Lineberger Comprehensive Cancer Center, The University of North Carolina at Chapel Hill, Chapel Hill, North Carolina, USA

[3] Department of Chemotherapy and Mycoses, National Institute of Infectious Diseases, Tokyo, Japan

[4] Department of Gastroenterology, Kanazawa University Graduate School of Medical Sciences, Kanazawa, Japan

[5] Cancer Research Institute, Kanazawa University, Kanazawa, Japan

[6] Institute for Frontier Science Initiative, Kanazawa University, Kanazawa, Japan

[7] Department of Endocrinology and Metabolism, Kanazawa University Graduate School of Medical Sciences, Kanazawa, Japan

[8] Department of Cellular and Molecular Function Analysis, Kanazawa University Graduate School of Medical Science, Kanazawa, Japan

[9] Department of Virology I, National Institute of Infectious Diseases, Tokyo, Japan

[10] Management Department of Biosafety and Laboratory Animal, National Institute of Infectious Diseases, Tokyo, Japan

[11] Department of Molecular Genetics, Kanazawa University Graduate School of Medical Science, Kanazawa, Japan

[12] Department of Biochemistry and Molecular Vascular Biology, Kanazawa University Graduate School of Medical Science, Kanazawa, Japan

[13] WPI-Nano Life Science Institute (WPI-NanoLSI), Kanazawa University, Kanazawa, Japan

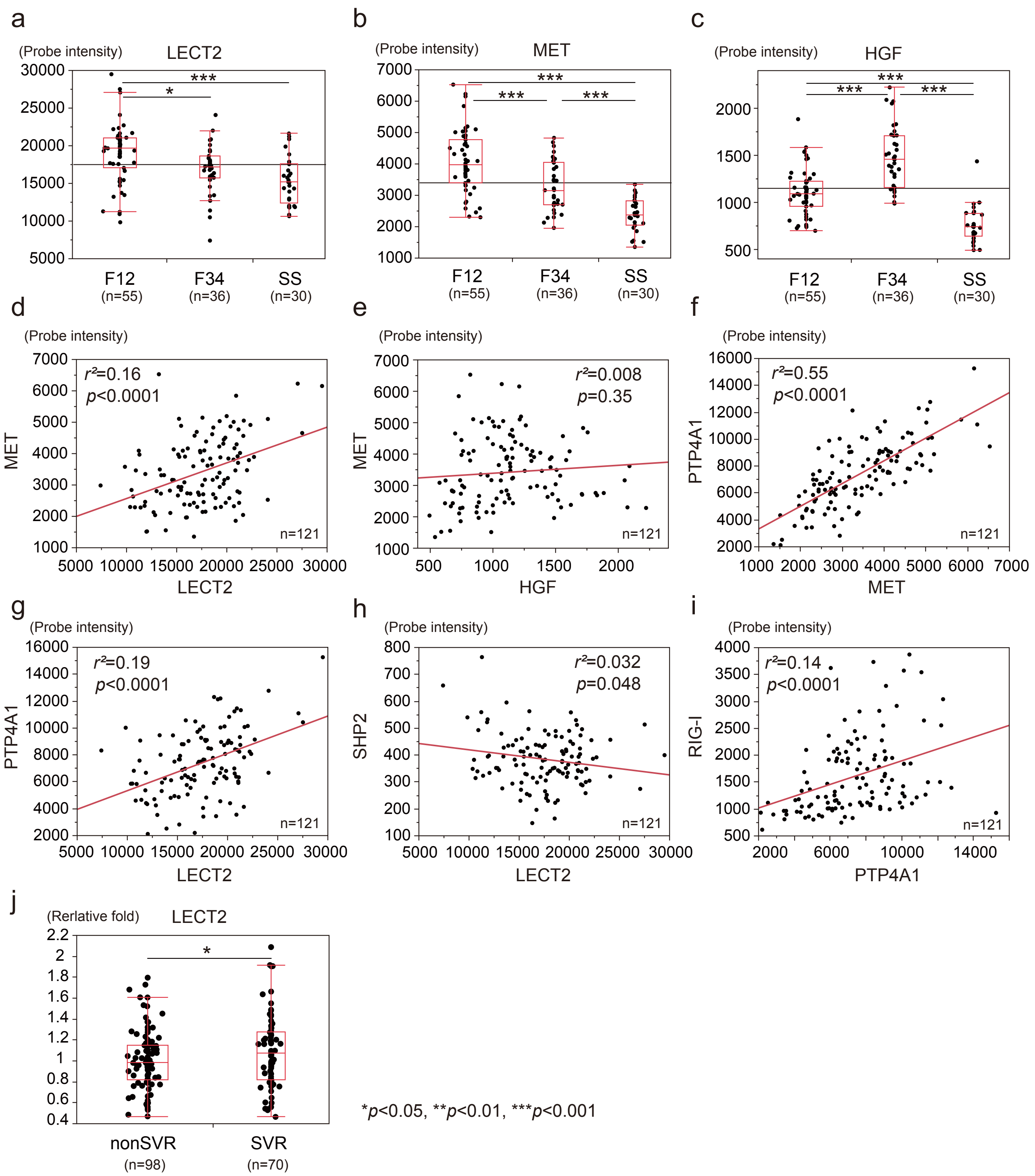

Supplementary Figure 1: mRNA levels in patients with chronic liver disease.

mRNA expression of *LECT2*, *MET*, *HGF*, *PTP4A1*, *SHP2* and *RIG-I* were evaluated using liver tissue obtained from 91 CHC patients (F12, n = 55; F34, n = 36) before they received PEG-

IFNa-2b and RBV combination therapy and 30 patients with early fibrosis (F12), advanced fibrosis (F34) and simple steatosis (SS). Boxplots: center line, median; box limits, 25 to 75th percentiles; whiskers, min to max. (**a-c**) Data shown are means ± SEM by two-way ANOVA with Tukey's multiple comparison test. (**d-i**) Simple linear regression analysis (one-sided) was performed between the two values. (**j**) Data shown are means ± SEM by two-way ANOVA with unpaired Student's t-test.

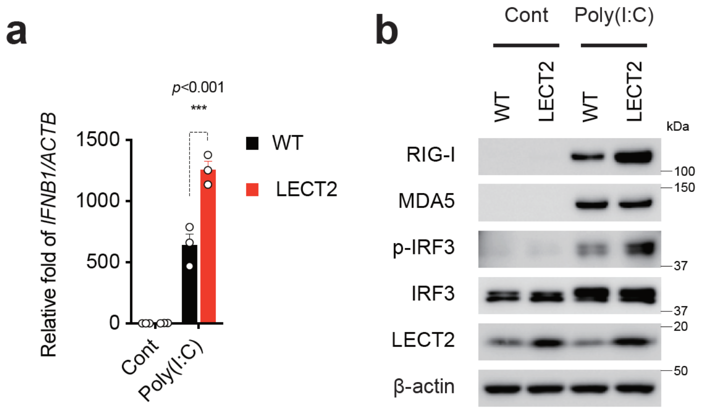

Supplementary Figure 2: LECT2 enhances innate immune responses induced by Poly(I:C). **(a, b)** HepG2-WT and HepG2-LECT2 cells were transfected with Poly(I:C). (**a**) qRT-PCR analysis of *IFNB1* in HepG2-WT and HepG2-LECT2 cells at 12 h after mock (Cont) or Poly(I:C). transfection. Results were normalised to those of *ACTB*. Data shown are means of 3 technical replicates ± SEM. ***$p < 0.001$ by two-way ANOVA with Tukey's multiple comparison test. (**b**) Immunoblot analysis of RIG-I, MDA5, p-IRF3, IRF3, LECT2, and β-actin in HepG2-WT and HepG2-LECT2 cells at 6 h after mock (Cont) or HCV-RNA transfection. Source data are provided as a Source Data file.

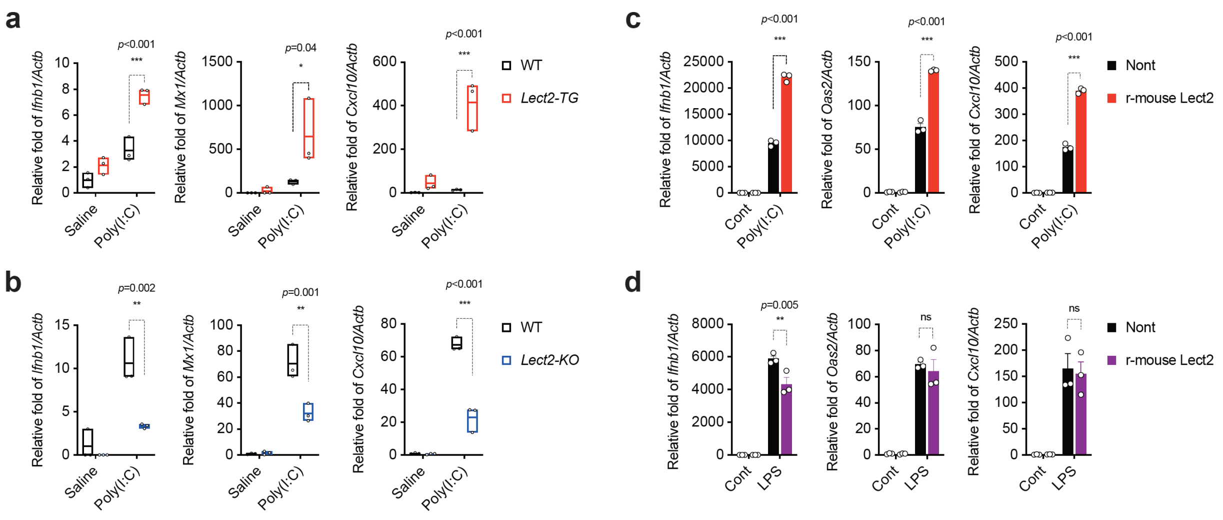

Supplementary Figure 3: Lect2 enhances innate immune responses induced by Poly(I:C) in mice and mouse primary hepatocytes.

(**a, b**) WT, *Lect2-TG*, and *Lect2-KO* mice were intravenously injected with saline or Poly(I:C). (**a**) qRT-PCR analysis of *Ifnb1*, *Mx1*, and *Cxcl10* in the liver of WT and *Lect2-TG* mice at 12 h after injection. Results were normalised to those of *Actb*. Data shown are means of 3 biological replicates ± SEM. ***p < 0.001, *p = 0.04 by two-way ANOVA with Tukey's multiple comparison test. Boxplots: center line, median; box limits, 25 to 75th percentiles; whiskers, min to max. (**b**) qRT-PCR analysis of *Ifnb1*, *Mx1*, and *Cxcl10* in the liver of WT and *Lect2-KO* mice at 12 h after injection. Results were normalised to those of *Actb*. Data shown are means of 3 biological replicates ± SEM. ***p < 0.001, **p = 0.002 (*Ifnb/Actb*), **p = 0.002 (*Mx1/Actb*) by two-way ANOVA with Tukey's multiple comparison test. Boxplots: center line, median; box limits, 25 to 75th percentiles; whiskers, min to max. (**c, d**) Mouse primary hepatocytes were treated with r-mouse Lect2 for 1 h and then transfected with Poly(I:C) or treated with LPS. (c) qRT-PCR analysis of *Ifnb1*, *Oas2*, and *Cxcl10* in cells at 12 h after mock (Cont) or Poly(I:C) transfection in the presence or absence (Nont) of r-mouse Lect2. Results were normalised to those of *Actb*. Data shown are means of 3 technical replicates ± SEM. ***p < 0.001 by two-way ANOVA with Tukey's multiple comparison test. (d) qRT-PCR analysis of Ifnb1, Oas2, and Cxcl10 in cells at 12 h after non-treatment (Cont) or LPS treatment in the presence or absence (Nont) of r-mouse Lect2. Results were normalised to those of *Actb*. Data shown are means of 3 technical replicates ± SEM. **p = 0.005, not significant (ns), by two-way ANOVA with Tukey's multiple comparison test. Source data are provided as a Source Data file.

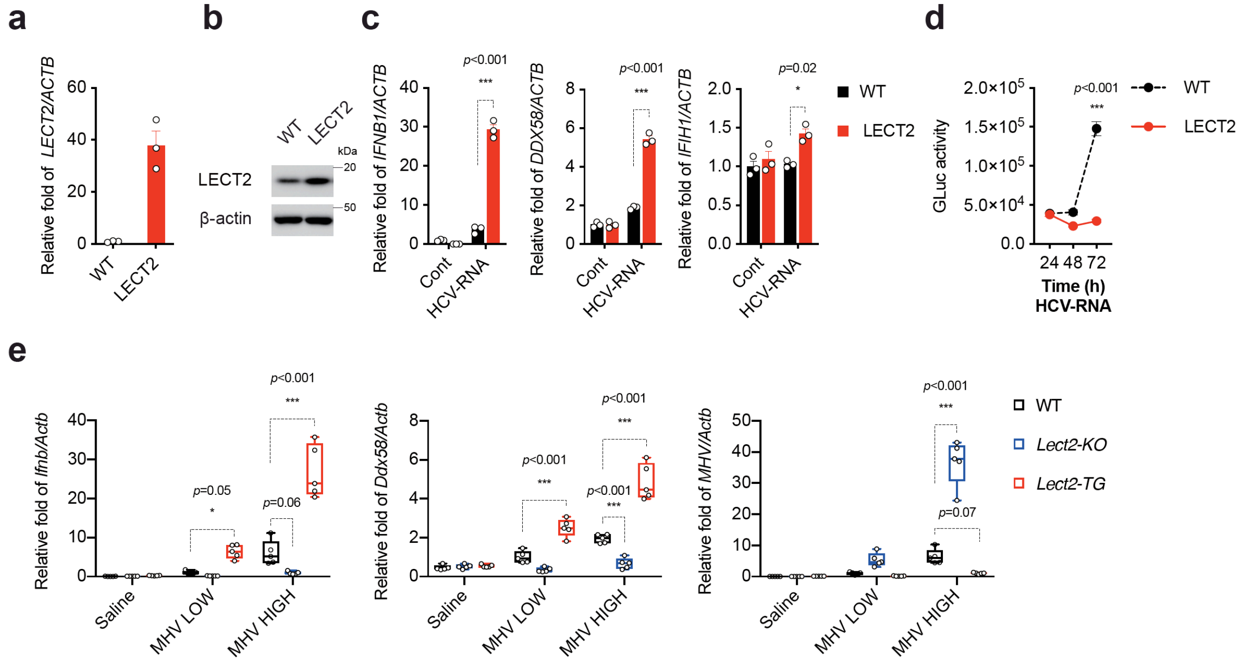

Supplementary Figure 4: LECT2 suppresses HCV and MHV replication.

(**a**) qRT-PCR analysis of *LECT2* in KH-WT and KH-LECT2 cells. Results were normalised to those of *ACTB*. (**b**) Immunoblot analysis of LECT2 and β-actin in KH-WT and KH-LECT2 cells. (**c, d**) KH-WT and KH-LECT2 cells were transfected with HCV-RNA (H77S.3/GLuc-RNA). (**c**) qRT-PCR analysis of *IFNB1*, *DDX58*, and *IFIH1* in KH-WT and KH-LECT2 cells at 12 h after mock (Cont) and HCV-RNA transfection. Results were normalised to those of *ACTB*. Data shown are means of 3 technical replicates ± SEM. ***p < 0.001, *p = 0.02 by two-way ANOVA with Tukey's multiple comparison test. (**d**) GLuc activity in KH-WT and KH-LECT2 cells at 12 h after HCV-RNA transfection. Data shown are means of 3 technical replicates ± SEM. ***p < 0.001 by two-way ANOVA with Tukey's multiple comparison test. (**e**) WT, *Lect2-TG*, and *Lect2-KO* mice were infected with 5 pfu (LOW) or 100 pfu (HIGH) MHV via intraperitoneal injection. qRT-PCR analysis of *Ifnb1*, *Ddx58*, and MHV in the liver of WT, *Lect2-TG*, and *Lect2-KO* mice at 7 days after saline injection or MHV infection. Results were normalised to those of *Actb*. Data shown are means of 5 biological replicates ± SEM. ***p < 0.001, *p = 0.05 by two-way ANOVA with Tukey's multiple comparison test. Boxplots: center line, median; box limits, 25 to 75th percentiles; whiskers, min to max. Source data are provided as a Source Data file.

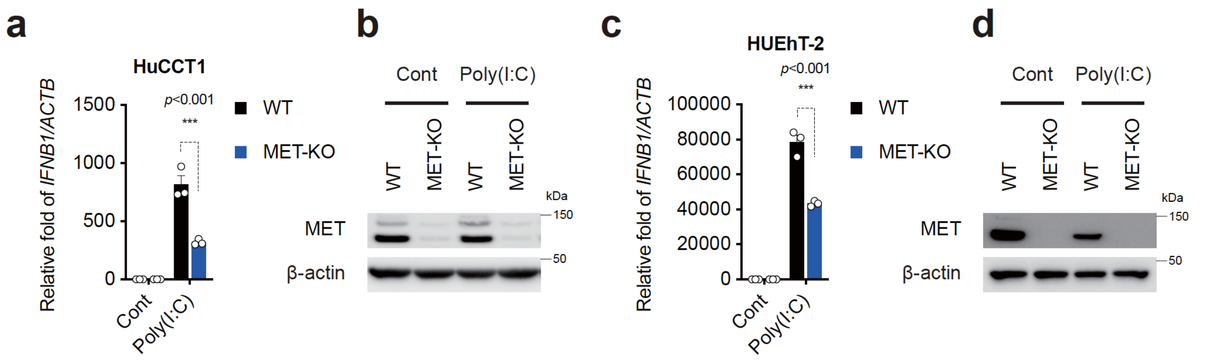

Supplementary Figure 5: MET has an important role in innate immune responses in HuCCT1 and HUEhT-2 cells.

(**a, b**) HuCCT1-WT and HuCCT1-MET-KO cells were transfected with Poly(I:C). (**a**) qRT-PCR analysis of *IFNB1* in HuCCT1-WT and HuCCT1-MET-KO cells at 12 h after mock (Cont) or Poly(I:C) transfection. Results were normalised to those of *ACTB*. Data shown are means of 3 technical replicates ± SEM. ***p < 0.001 by two-way ANOVA with Tukey's multiple comparison test. (**b**) Immunoblot analysis of MET and β-actin in HuCCT1-WT and HuCCT1-MET-KO cells at 12 h after mock (Cont) or Poly(I:C) transfection. (**c, d**) HUEhT-2-WT and HUEhT-2-MET-KO cells were transfected with Poly(I:C). (**c**) qRT-PCR analysis of *IFNB1* in HUEhT-2-WT and HUEhT-2-MET-KO cells at 12 h after mock (Cont) or Poly(I:C) transfection. Results were normalised to those of *ACTB*. Data shown are means of 3 technical replicates ± SEM. ***p < 0.001 by two-way ANOVA with Tukey's multiple comparison test. (**d**) Immunoblot analysis of MET and β-actin in HUEhT-2-WT and HUEhT-2-MET-KO cells at 12 h after mock (Cont) or Poly(I:C) transfection. Source data are provided as a Source Data file.

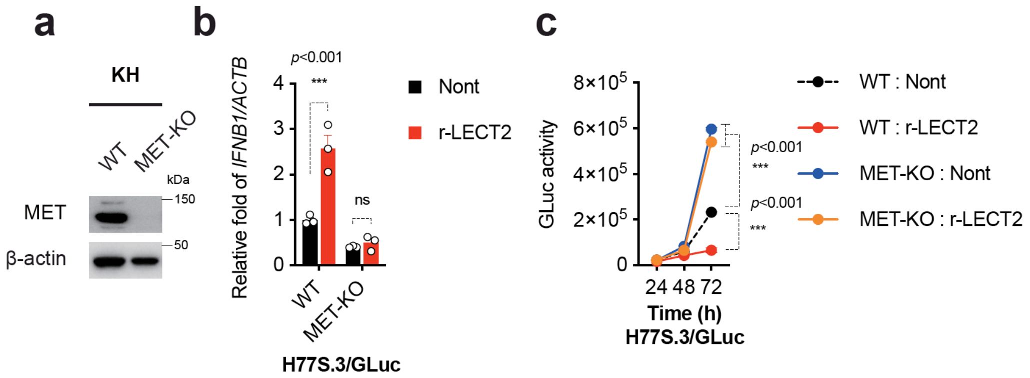

Supplementary Figure 6: MET is essential for LECT2-mediated innate immune responses in KH cells.

(**a**) Immunoblot analysis of MET and β-actin in KH-WT and KH-MET-KO cells. (**b, c**) KH-WT and KH-MET-KO cells were treated with r-LECT2 at a concentration of 50 ng/mL for 1 h and then transfected with H77S.3/GLuc-RNA. (**b**) qRT-PCR analysis of *IFNB1* in KH-WT and KH-MET-KO cells at 12 h after H77S.3/GLuc-RNA transfection in the presence or absence (Nont) of r-LECT2. Results were normalised to those of *ACTB*. Data shown are means of 3 technical replicates ± SEM. ***$p < 0.001$, not significant (ns) by two-way ANOVA with Tukey's multiple comparison test. (**c**) GLuc activity in KH-WT and KH-MET-KO cells at 24, 48, and 72 h after H77S.3/GLuc-RNA transfection in the presence or absence (Nont) of r-LECT2. Data shown are means of 3 technical replicates ± SEM. ***$p < 0.001$ by two-way ANOVA with Tukey's multiple comparison test. Source data are provided as a Source Data file.

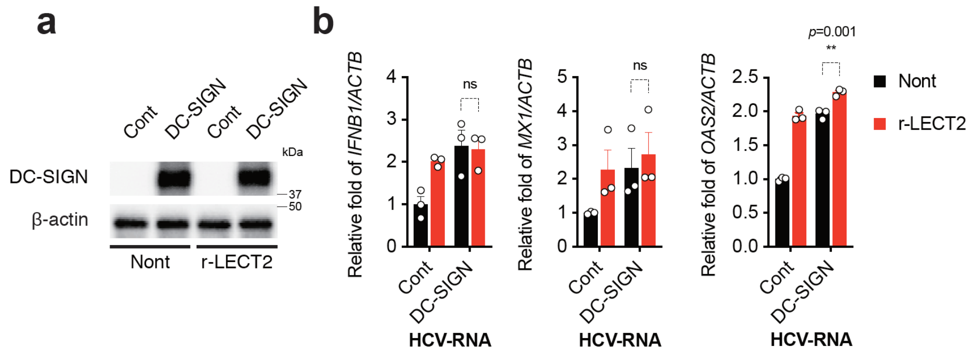

Supplementary Figure 7: DC-SIGN does not affect LECT2-mediated innate immune responses.

(**a, b**) HepG2 cells were transfected with the DC-SIGN plasmid. At 48 h after transfection, the cells were treated with 50 ng/mL r-LECT2 for 1 h and then transfected with HCV-RNA. (**a**) Immunoblot analysis of DC-SIGN and β-actin in these cells at 12 h after HCV-RNA transfection in the presence or absence (Nont) of r-LECT2. (**b**) qRT-PCR analysis of *IFNB1*, *MX1*, and *OAS2* in in these cells at 12 h after HCV-RNA transfection in the presence or absence (Nont) of r-LECT2. Results were normalised to those of *ACTB*. Data shown are means of 3 technical replicates ± SEM. $**p = 0.001$, not significant (ns) by two-way ANOVA with Tukey's multiple comparison test. Source data are provided as a Source Data file.

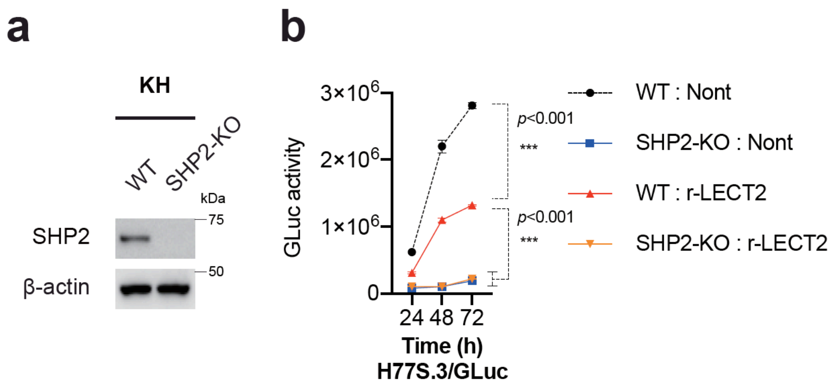

Supplementary Figure 8: LECT2 does not affect HCV replication in the absence of SHP2. **(a)** Immunoblot analysis of SHP2 and β-actin in KH-WT and KH-SHP2-KO cells. (**b**) KH-WT and KH-SHP2-KO cells were treated with 50 ng/mL r-LECT2 for 1 h and then transfected with H77S.3/GLuc-RNA. GLuc activity in KH-WT and KH-SHP2-KO cells at 24, 48, and 72 h after H77S.3/GLuc-RNA transfection in the presence or absence (Nont) of r-LECT2. Data shown are means of 3 technical replicates ± SEM. ***p < 0.001 by two-way ANOVA with Tukey's multiple comparison test. Source data are provided as a Source Data file.

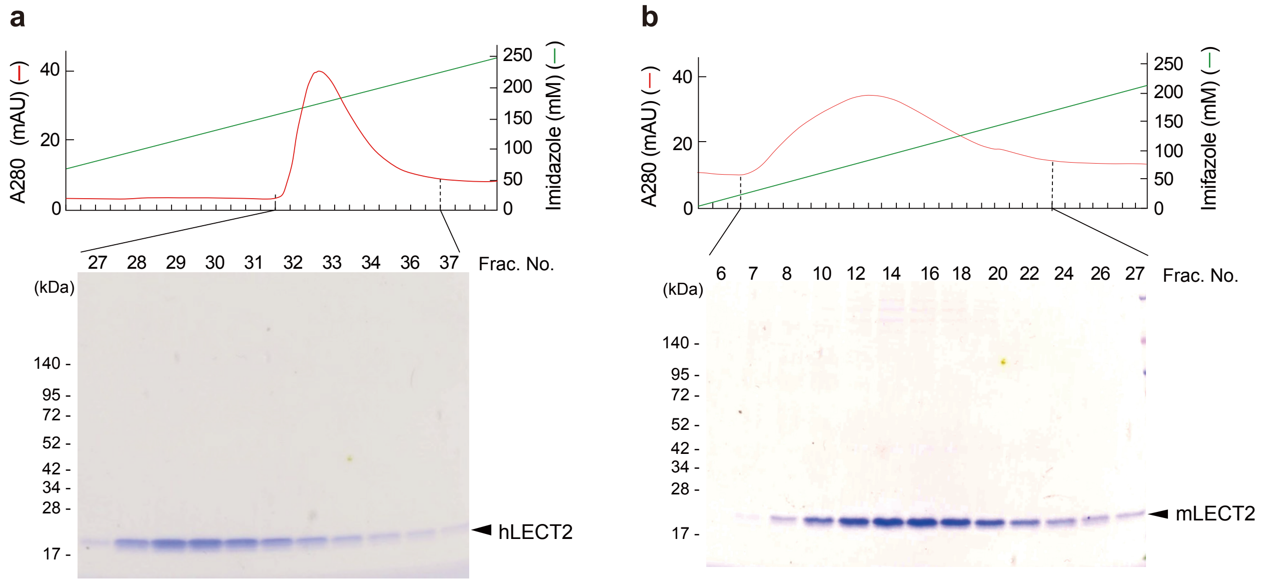

Supplementary Figure 9: Preparation of recombinant LECT2.

(**a, b; upper**) Human and mouse recombinant LECT2 were eluted using a concentration gradient of imidazole. Fractions containing LECT2 were applied and eluted using a heparin column to remove imidazole. (**a, b; lower**) Coomassie brilliant blue staining for recombinant LECT2 in each fraction.

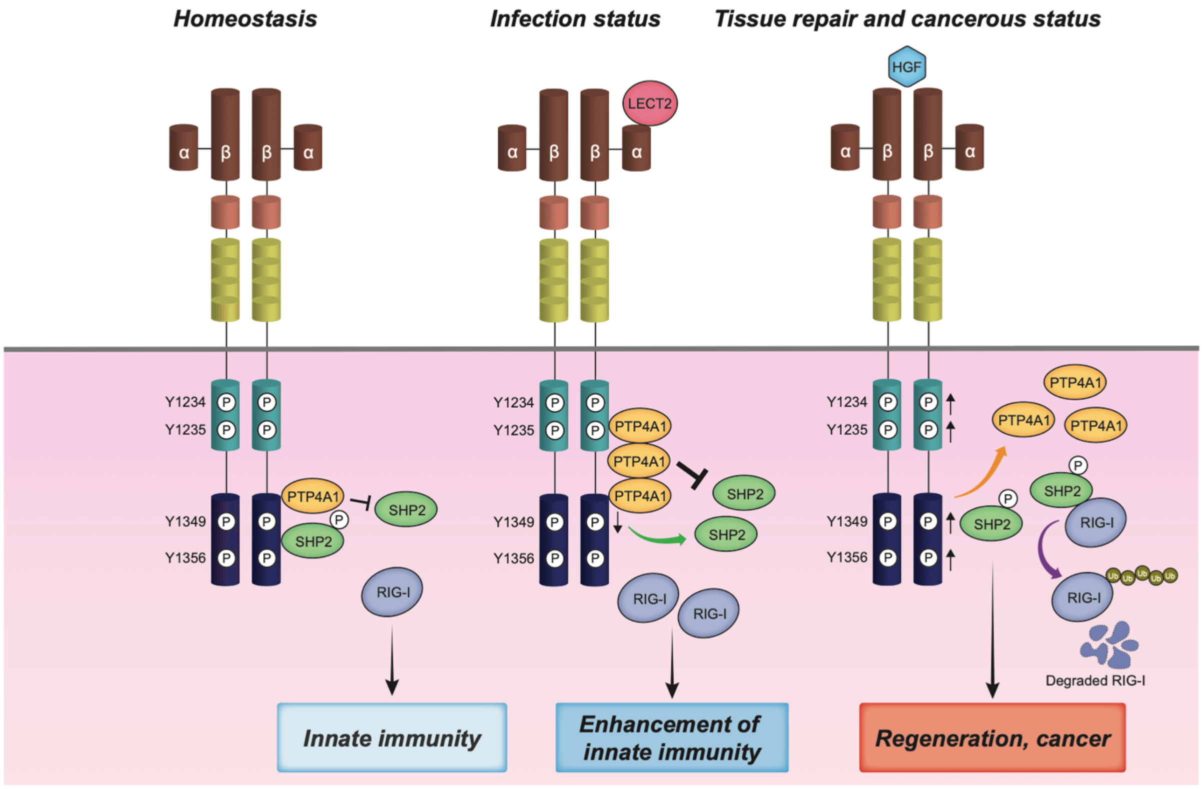

Supplementary Figure 10: Schematic representation of the role of LECT2 in innate immune response. LECT2 promotes the recruitment of PTP4A1 to MET, and PTP4A1 dephosphorylates SHP2 under ligand-free conditions. PTP4A1 and SHP2 have antagonistic effects on MET, and their competition for binding MET maintains a normal homeostatic balance. When cells are infected with virus, LECT2 becomes the predominant ligand for MET, tipping the balance toward PTP4A1 engagement and resulting in an enhancement of RIG-I responses. In contrast, in tissue repair and cancerous states, HGF is the predominant ligand for MET, resulting in a suppression of the innate immune response and enhancement of proliferative signals.
