# Supplementary material for: Leukocyte cell-derived chemotaxin 2 is an antiviral regulator acting through the proto-oncogene MET
Source: Nat Commun. 2022 Jun 8;13:3176. doi: 10.1038/s41467-022-30879-3 (PMC9177837; doi:10.1038/s41467-022-30879-3)
Supplement: Supplementary file 3 — Reporting Summary [file 41467_2022_30879_MOESM3_ESM.pdf]

Corresponding author(s): Takayoshi Shirasaki, Masao Honda

Last updated by author(s): 5/12/2022

Reporting Summary

Nature Research wishes to improve the reproducibility of the work that we publish. This form provides structure for consistency and transparency in reporting. For further information on Nature Research policies, see our Editorial Policies and the Editorial Policy Checklist.

Statistics

For all statistical analyses, confirm that the following items are present in the figure legend, table legend, main text, or Methods section.

n/a	Confirmed	
☐	☒	The exact sample size (*n*) for each experimental group/condition, given as a discrete number and unit of measurement
☐	☒	A statement on whether measurements were taken from distinct samples or whether the same sample was measured repeatedly
☐	☒	The statistical test(s) used AND whether they are one- or two-sided<br>*Only common tests should be described solely by name; describe more complex techniques in the Methods section.*
☒	☐	A description of all covariates tested
☐	☒	A description of any assumptions or corrections, such as tests of normality and adjustment for multiple comparisons
☐	☒	A full description of the statistical parameters including central tendency (e.g. means) or other basic estimates (e.g. regression coefficient) AND variation (e.g. standard deviation) or associated estimates of uncertainty (e.g. confidence intervals)
☐	☒	For null hypothesis testing, the test statistic (e.g. *F*, *t*, *r*) with confidence intervals, effect sizes, degrees of freedom and *P* value noted<br>*Give P values as exact values whenever suitable.*
☒	☐	For Bayesian analysis, information on the choice of priors and Markov chain Monte Carlo settings
☒	☐	For hierarchical and complex designs, identification of the appropriate level for tests and full reporting of outcomes
☐	☒	Estimates of effect sizes (e.g. Cohen's *d*, Pearson's *r*), indicating how they were calculated

Our web collection on statistics for biologists contains articles on many of the points above.

Software and code

Policy information about availability of computer code

Data collection	Immuno Blots were acquired from ChemiDoc Imaging System with Image Lab 6.0 software (Bio-Rad). Image were acquired from BZ-9000 microscope (KEYENCE) or Dragonfly CR-DFLY-301 (Oxford Instruments, Oxon UK). Luciferase activity was measured with GloMax-Multi Detection System (Promega). qPCR data was acquired on 7500 Real Time PCR System (Applied Biosystems).
Data analysis	Statistical analysis was performed using GraphPad Prism 8.

For manuscripts utilizing custom algorithms or software that are central to the research but not yet described in published literature, software must be made available to editors and reviewers. We strongly encourage code deposition in a community repository (e.g. GitHub). See the Nature Research guidelines for submitting code & software for further information.

Data

Policy information about availability of data

All manuscripts must include a data availability statement. This statement should provide the following information, where applicable:

- Accession codes, unique identifiers, or web links for publicly available datasets
- A list of figures that have associated raw data
- A description of any restrictions on data availability

All data are available in the manuscript, in the Supplementary Information or in the Source Data files.

Field-specific reporting

Please select the one below that is the best fit for your research. If you are not sure, read the appropriate sections before making your selection.

- [x] Life sciences
- [ ] Behavioural & social sciences
- [ ] Ecological, evolutionary & environmental sciences

For a reference copy of the document with all sections, see nature.com/documents/nr-reporting-summary-flat.pdf

Life sciences study design

All studies must disclose on these points even when the disclosure is negative.

Sample size	No sample size calculations were performed. Sample sizes were determined to be adequate based on the magnitude and consistency of measurable differences between groups. The number of experiments or samples is indicated in each figure legend.
Data exclusions	No data were excluded from the analyses.
Replication	Data reproducibility was confirmed in multiple independent experiments as described in legends.
Randomization	For in vitro experiments, samples were randomly assigned. For in vivo experiments, Age- and sex-matched wild-type, Lect2-TG and Lect2-KO mice were randomly allocated.
Blinding	The investigators were blinded during the experiments, and during which only numbers were used to define samples.

Reporting for specific materials, systems and methods

We require information from authors about some types of materials, experimental systems and methods used in many studies. Here, indicate whether each material, system or method listed is relevant to your study. If you are not sure if a list item applies to your research, read the appropriate section before selecting a response.

Materials & experimental systems

n/a	Involved in the study	
[ ]	[x]	Antibodies
[ ]	[x]	Eukaryotic cell lines
[x]	[ ]	Palaeontology and archaeology
[ ]	[x]	Animals and other organisms
[ ]	[x]	Human research participants
[x]	[ ]	Clinical data
[x]	[ ]	Dual use research of concern

Methods

n/a	Involved in the study	
[x]	[ ]	ChIP-seq
[x]	[ ]	Flow cytometry
[x]	[ ]	MRI-based neuroimaging

Antibodies

Antibodies used	Primary antibodies:<br>RIG-I (#3743, 1:1000), MDA5 (#5321, 1:1000), p-IRF3 (#83611, 1:1000), IRF3 (#4302, 1:1000), p-MET (Tyr1234/1235) (#3077, 1:1000), p-MET (Tyr1349) (#3121, 1:1000), MET (#3127, 1:1000), p-GAB1 (#12745, 1:1000), GAB1 (#3232, 1:1000), p-SHP2 (#5431, 1:1000), SHP2 (#3397, 1:1000), p-ERK (#9101, 1:1000), ERK1/2 (#9102, 1:1000), DC-SIGN (#13193, 1:1000), DYKDDDDK Tag (FLAG) (#14793, 1:1000), c-Cbl (#2747, 1:1000), Myc (#2276, 1:1000), GAPDH (#2118, 1:1000), and b-actin (ACTB; #4970, 1:1000) were purchased from Cell Signaling Technology (Beverly, MA); phosphotyrosine (05-321, 1:1000) and K48-ubiquitin (05-1307, 1:1000) were from Merck Millipore (Burlington, MA); PRL-1 (PTP4A1) (ab168643, 1:1000) and LECT2 (ab119429, 1:1000) were from Abcam (Cambridge, UK); and p-MET (Tyr1356) (PA5-40218, 1:1000) was from Thermo Fisher Scientific.<br>Secondary antibodies:<br>Anti-rabbit IgG, HRP-linked Antibody (#7074, 1:1000) and Anti-mouse IgG, HRP-linked Antibody (#7076) were purchased from Cell Signaling Technology; goat anti-rabbit Alexa Fluor 488 (A27034, 1:1000), goat anti-rabbit Alexa Fluor 594 (A11012, 1:1000), goat anti-mouse Alexa Fluor 488 (A28175, 1:1000) and goat anti-mouse Alexa Fluor 594 (A11032, 1:1000) were purchased from Invitrogen.
Validation	All the commercial antibodies have been verified by the manufactures.<br>Cell Signaling:<br>RIG-I, https://www.cellsignal.com/products/primary-antibodies/rig-i-d14g6-rabbit-mab/3743<br>MDA5, https://www.cellsignal.com/products/primary-antibodies/mda-5-d74e4-rabbit-mab/5321<br>p-IRF3, https://www.cellsignal.com/products/antibody-conjugates/phospho-irf-3-ser396-d6o1m-rabbit-mab-pe-conjugate/83611<br>IRF3, https://www.cellsignal.com/products/primary-antibodies/irf-3-d83b9-rabbit-mab/4302<br>p-MET (Tyr1234/1235), https://www.cellsignal.com/products/primary-antibodies/phospho-met-tyr1234-1235-d26-xp-rabbit-mab/3077<br>p-MET (Tyr1349), https://www.cellsignal.com/products/primary-antibodies/phospho-met-tyr1349-antibody/3121

April 2020

MET, https://www.cellsignal.com/products/primary-antibodies/met-25h2-mouse-mab/3127
p-GAB1, https://www.cellsignal.com/products/primary-antibodies/phospho-gab1-tyr659-antibody/12745
GAB1, https://www.cellsignal.com/products/primary-antibodies/gab1-antibody/3232
p-SHP2, https://www.cellsignal.com/products/primary-antibodies/phospho-shp-2-tyr580-d66f10-rabbit-mab/5431
SHP2, https://www.cellsignal.com/products/primary-antibodies/shp-2-d50f2-rabbit-mab/3397
p-ERK, https://www.cellsignal.com/products/primary-antibodies/phospho-p44-42-mapk-erk1-2-thr202-tyr204-antibody/9101
ERK, https://www.cellsignal.com/products/primary-antibodies/p44-42-mapk-erk1-2-antibody/9102
DC-SING, https://www.cellsignal.com/products/primary-antibodies/dc-sign-d7f5c-xp-rabbit-mab/13193
DYKDDDDK Tag, https://www.cellsignal.com/products/primary-antibodies/dykddddk-tag-d6w5b-rabbit-mab-binds-to-same-epitope-as-sigma-s-anti-flag-m2-antibody/14793
c-Cbl, https://www.cellsignal.com/products/primary-antibodies/c-cbl-antibody/2747
Myc, https://www.cellsignal.com/products/primary-antibodies/myc-tag-9b11-mouse-mab/2276
GAPDH, https://www.cellsignal.com/products/primary-antibodies/gapdh-14c10-rabbit-mab/2118
b-actin, https://www.cellsignal.com/products/primary-antibodies/b-actin-13e5-rabbit-mab/4970
Anti-rabbit IgG, HRP-linked Antibody, https://www.cellsignal.com/products/secondary-antibodies/anti-rabbit-igg-hrp-linked-antibody/7074
Anti-mouse IgG, HRP-linked Antibody, https://www.cellsignal.com/products/secondary-antibodies/anti-mouse-igg-hrp-linked-antibody/7076
Merck Millipore:
phosphotyrosine, https://www.emdmillipore.com/US/en/product/Anti-Phosphotyrosine-Antibody-clone-4G10,MM_NF-05-321
K48-ubiquitin, https://www.emdmillipore.com/US/en/product/Anti-Ubiquitin-Antibody-Lys48-Specific-clone-Apu2-rabbit-monoclonal,MM_NF-05-1307
abcam:
PRL-1, https://www.abcam.com/prl-1-antibody-ab168643.html
LECT2, https://www.abcam.com/lect2-antibody-oti2a11-ab119429.html
Thermo Fisher Scientific:
p-MET (Tyr1356), https://www.thermofisher.com/antibody/product/Phospho-c-Met-Tyr1356-Antibody-Polyclonal/PA5-40218
Invitrogen:
goat anti-rabbit Alexa Fluor 488, https://www.thermofisher.com/antibody/product/Goat-anti-Rabbit-IgG-H-L-Secondary-Antibody-Recombinant-Polyclonal/A27034

Eukaryotic cell lines

Policy information about cell lines

Cell line source(s)	HepG2 cells were purchased from the American Type Culture Collection (Manassas, VA). KH cells were established from surgically resected hepatocellular carcinoma tissue from a patient with CHC. HuCCT1 cells and HUEhT-2 cells were purchased from JCRB (Japanese Collection of Research Bioresources) Cell Bank (National Institutes of Biomedical Innovation, Health and Nutrition, Osaka, Japan). Lenti-X 293T cells were purchased from Takara Bio.
Authentication	Cell lines were confirmed by immunoblotting of proteins and RTD-PCR of gene expression.
Mycoplasma contamination	Cell lines were tested for mycoplasma contamination by a PCR-based method and found to be negative.
Commonly misidentified lines (See ICLAC register)	No commonly misidentified cell lines were used in this study.

Animals and other organisms

Policy information about studies involving animals; ARRIVE guidelines recommended for reporting animal research

Laboratory animals	6-8 week old, male, wild type C57BL/6, Lect2-TG and Lect2-KO mice were used in this study. The mice were kept in a controlled environment with 40% humidity, 20°C temperature conditions and 12h light-and-dark cycles, and were accessible at all times to water and food. All mice were bred and kept under specific pathogen-free conditions.
Wild animals	No wild animals were used in this study.
Field-collected samples	The study did not involve field-collected samples.
Ethics oversight	All mouse studies were carried out in accordance with the Guidelines for the Care and Use of Laboratory Animals issued by the National Institute of Infectious Diseases. The protocol was approved by the ethics committee of the National Institute of Infectious Diseases.

Note that full information on the approval of the study protocol must also be provided in the manuscript.

Human research participants

Policy information about studies involving human research participants

Population characteristics	mRNA expression was evaluated using liver tissue obtained from 91 (median age=56, M:F=57:34) CHC patients (F12, n = 55; F34, n = 36) before they received PEG-IFNa-2b (Schering-Plough K.K.) and RBV combination therapy, and 30 patients with SS. Patients characteristics were summarized in previously reported (Cell Host Microbe. 2019 25(4):588-601).
Recruitment	Among the three groups of patients, namely, CHC(F12), CHC(F34), and SS, the age of SS was significantly lower than that of CHC(F12+F34). BMI and T-Chol serum levels were significantly higher in SS than in CHC(F12+F34). ALT and g-GTP levels were significantly higher in CHC(F34) than in SS and CHC(F12). The PLT count was significantly lower in CHC(F34) than in SS and CHC(F12). Patients characteristics were summarized in previously reported (Cell Host Microbe. 2019 25(4):588-601).
Ethics oversight	The research protocols were reviewed by the ethics committee at Kanazawa University and its related hospitals. Informed consent was obtained from all patients. No compensation was applied for this study.

Note that full information on the approval of the study protocol must also be provided in the manuscript.
